# Interplay of chronic obstructive pulmonary disease and colorectal cancer development: unravelling the mediating role of fatty acids through a comprehensive multi-omics analysis

**DOI:** 10.1186/s12967-023-04278-1

**Published:** 2023-09-01

**Authors:** Youtao Zhou, Zikai Lin, Shuojia Xie, Yuan Gao, Haobin Zhou, Fengzhen Chen, Yuewu Fu, Cuiyan Yang, Chuanfeng Ke

**Affiliations:** 1 The First Clinical Medical School, Guangzhou Medical University, Guangzhou, China; 2 Nanshan School, Guangzhou Medical University, Guangzhou, China; 3 Department of General Surgery, School of Medicine, The First Affiliated Hospital, Ji’nan University, Guangzhou, China; 4 Department of Gastrointestinal Surgery, The First Affiliated Hospital of Guangzhou Medical University, Guangzhou, China

**Keywords:** Chronic obstructive pulmonary disease, Colorectal cancer, Fatty acid, Multi-omics, Mediating factor

## Abstract

**Background:**

Chronic obstructive pulmonary disease (COPD) patients often exhibit gastrointestinal symptoms, A potential association between COPD and Colorectal Cancer (CRC) has been indicated, warranting further examination.

**Methods:**

In this study, we collected COPD and CRC data from the National Health and Nutrition Examination Survey, genome-wide association studies, and RNA sequence for a comprehensive analysis. We used weighted logistic regression to explore the association between COPD and CRC incidence risk. Mendelian randomization analysis was performed to assess the causal relationship between COPD and CRC, and cross-phenotype meta-analysis was conducted to pinpoint crucial loci. Multivariable mendelian randomization was used to uncover mediating factors connecting the two diseases. Our results were validated using both NHANES and GEO databases.

**Results:**

In our analysis of the NHANES dataset, we identified COPD as a significant contributing factor to CRC development. MR analysis revealed that COPD increased the risk of CRC onset and progression (OR: 1.16, 95% CI 1.01–1.36). Cross-phenotype meta-analysis identified four critical genes associated with both CRC and COPD. Multivariable Mendelian randomization suggested body fat percentage, omega-3, omega-6, and the omega-3 to omega-6 ratio as potential mediating factors for both diseases, a finding consistent with the NHANES dataset. Further, the interrelation between fatty acid-related modules in COPD and CRC was demonstrated via weighted gene co-expression network analysis and Kyoto Encyclopedia of Genes and Genomes enrichment results using RNA expression data.

**Conclusions:**

This study provides novel insights into the interplay between COPD and CRC, highlighting the potential impact of COPD on the development of CRC. The identification of shared genes and mediating factors related to fatty acid metabolism deepens our understanding of the underlying mechanisms connecting these two diseases.

**Supplementary Information:**

The online version contains supplementary material available at 10.1186/s12967-023-04278-1.

## Introduction

In the global cancer statistics provided by GLOBOCAN in 2020, colorectal cancer (CRC) ranks as the third most commonly diagnosed cancer and the second leading cause of cancer-related deaths worldwide, with an incidence rate of 10.0% and a mortality rate of 9.4% [1]. Patients with CRC exhibit differences in symptoms and prognosis, and genetic heterogeneity is believed to be of significant importance for the treatment and survival of CRC patients such as microsatellite instability and chromosomal instability [2].

Numerous factors influence the onset of CRC and patient survival, and chronic obstructive pulmonary disease (COPD) was identified as a potential factor in the progression of CRC [3]. In 2019, approximately 391.9 million individuals 30–79 years old were reported with COPD worldwide, posing a substantial burden on global healthcare systems [4]. COPD progression involves a complex interplay between multiple genetic and epigenetic components in addition to diverse environmental factors [5]. COPD patients often exhibit multifaceted progressions and a wide array of comorbidities, which can be attributed to the multitude of contributing factors. Gastrointestinal dysfunction is commonly associated with COPD [6]. However, despite the prevalence of gastrointestinal symptoms among COPD patients, such as the possible activity limitations in domestic routines due to repeated intestinal cell damage in COPD patients, this phenomenon has largely been overlooked in clinical settings [7]. A growing body of research has unveiled correlations between the progression or mortality of COPD and CRC. One nationwide retrospective cohort study revealed that CRC patients with COPD had an increased risk of mortality [8]. Additionally, a prospective follow-up investigation of CRC patients demonstrated significant associations between COPD and comorbidities such as diabetes and cardiocerebrovascular disease [9]. Previous observational studies have provided limited evidence supporting a causal relationship between COPD and CRC. Therefore, there is an urgent need to bolster these findings and identify potential underlying mechanisms.

As a chronic inflammatory condition, COPD is often accompanied by elevated levels of pro-inflammatory cytokines [10, 11]. When these cytokines enter the gastrointestinal tract, they promote changes in metabolic products, reflecting the gut-lung axis’s function [12]. In both lung epithelial cells of COPD patients and tumour cells in CRC patients, perturbations in lipid metabolism products have a critical role in the growth of tumor cells [13, 14]. For example, omega-3 and omega-6 fatty acids have been implicated in modulating persistent inflammation in COPD patients [15]. The ratio of omega-3 and omega-6 fatty acids influences rectal cell proliferation in CRC patients, with deviations from a standard ratio leading to suboptimal outcomes [16]. However, one study that analysed 11 common malignancies posited contradictory conclusions, suggesting that omega-3 fatty acids do not reduce the risk of developing cancer [9]. Whether omega-3 and omega-6 fatty acids exert causal effects on the metabolism of both fatty acid types in COPD and CRC patients is still unknown. The potential interplay between fatty acids and the gut-lung axis in connecting COPD and CRC remains to be clarified.

Multi-omics analysis involves the application of two or more omics methodologies to investigate genes and their expression products, thereby enhancing our understanding of the intricate relationships among diseases [17]. Mendelian randomization (MR) is as a valuable tool for inferring causal relationships between diseases by leveraging data obtained from genome-wide association studies (GWAS) [18]. Through bioinformatics analyses of transcriptomic data, researchers can examine disease-associated gene expression products; these results help shed light on the interconnectivity of disease pathways and the shared regulatory gene networks between various diseases [19].

In this study, we used a comprehensive approach by incorporating clinical examination data, GWAS, and transcriptomic data to examine the relationship between COPD and CRC. We further explored potential mediating factors including omega-3 and omega-6 fatty acids that may influence this association.

## Methods

### Literature search

We conducted a literature search on PubMed from 1963 to 2023 using the keywords “Chronic Obstructive Pulmonary Disease” and “Colorectal Cancer”, aiming to discover the established relationship between COPD and CRC as demonstrated by previous researchers.

### Data acquisition

The National Health and Nutrition Examination Survey (NHANES) is based on a complex multi-stage sampling weighting design to obtain a representative sample of the non-institutionalised USA civilian population and is designed to assess the health and nutritional status of the non-institutionalised USA population (https://wwwn.cdc.gov/nchs/nhanes/) [20]. Our analysis included participants who participated in NHANES in the 2003–2004 cycle. Participants who did not have fatty acid measurements and disease diagnosis were excluded, resulting in the inclusion of 1729 participants in the final analysis (Additional file 1: Fig. S1). NHANES was approved by the US Centers for Disease Control and Prevention (CDC) National Center for Health Statistics Institutional Review Board. Informed consent was obtained from all participants [21].

A summary of the COPD data was obtained from a case–control GWAS meta-analysis from the Global Biobank Meta-analysis Initiative (GBMI) [22]. The combined sample size from all discovery studies was 81,568 cases and 1,310,798 controls, spanning individuals of European (EUR), African (AFR), admixed American (AMR), East Asian (EAS), Middle Eastern (MID), and Central and South Asian (CSA) ancestry. Further information regarding the GBMI cohort can be found at the following website: https://www.globalbiobankmeta.org/. A GWAS of CRC was conducted among 64,190 individuals [23]. Whole-genome sequencing (WGS) data were obtained from 1439 CRC cases and 720 controls from 5 studies, and GWAS array data were obtained from 58,131 CRC or advanced adenoma cases (3674; 6.3% of cases) and 67,347 controls from 45 studies conducted by the Genetic Epidemiology of Colorectal Cancer Consortium (GECCO), Colorectal Cancer Family Registry (CCFR), and Colon Cancer Family Registry (CORECT). We used a Phase I meta-analysis consisting of existing genotyping data from 30 studies with 34,869 cases and 29,051 controls. We first collected genome-wide association data for omega-3 fatty acids from the open GWAS database. We selected two phenotypes, namely omega-3 fatty acids and the ratio of omega-3 fatty acids to total fatty acids. The study was jointly conducted by Nightingale Health and the UK Biobank, with 114,999 participants’ data included. The data and detailed descriptions can be found at https://gwas.mrcieu.ac.uk/.

The transcriptome and clinical information of patients with CRC and COPD were downloaded from GEO databases (https://www.ncbi.nlm.nih.gov/geo/). The GEO CRC cohorts included GSE15781 (n = 42), GSE17536 (n = 177) and GSE29621 (n = 65) datasets. The GEO COPD cohort was GSE57148 (n = 189) (https://www.ncbi.nlm.nih.gov/geo/).

### Definition of COPD and colon cancer in NHANES

COPD was defined as the “mcq160g” information from the “mcq” questionnaire in the NHANES data, which was labelled as “informed by hospital of the presence of emphysema [24].” Colon cancer was defined as the “mcq230” information from the “mcq” questionnaire in the NHANES data, which was labelled as “What kind of cancer did you have?” with the answer of colon cancer.

### Determination of omega-3 fatty acid and omega-6 fatty acids

NHANES uses fatty acid assays to measure the concentration of fatty acids in human blood using electron capture negative ion mass spectrometry. Omega-3 fatty acids include alpha-linolenic acid, eicosapentaenoic acid, and docosahexaenoic acid, while omega-6 fatty acids include linoleic acid and arachidonic acid [25]. We defined the total concentration of the two Omega fatty acids as the sum of the concentrations of their main types. The ratio of overall omega-6 fatty acids to overall omega-3 fatty acids was defined as the omega-6/omega-3 ratio.

### MR analysis

We conducted a MR analysis to investigate the associations between genome-wide significant single nucleotide polymorphisms (SNPs) and various phenotypes. For each cohort, we extracted SNPs with a P-value less than 5 × 10^−8^, considering them as significant variants associated with the phenotype for subsequent analyses [26]. To account for linkage disequilibrium (LD), we applied an LD exclusion criterion with an R2 threshold of 0.001 and a maximum distance threshold of 10,000 kilobases (kb) [27]. In instances where SNPs were found to be in LD, we selected the SNP with the lowest P-value for further analysis. To mitigate the potential impact of weak instrument bias, we calculated the F-statistic for each SNP and excluded SNPs with an F-statistic less than 10, because such SNPs were considered weak instrumental variables that could introduce bias into the results [28].

For the MR analysis, we primarily employed the inverse variance weighting (IVW) method. In cases where only one instrumental variable was present, the Wald Ratio was used to estimate the effect of exposure on the outcome. We also performed a leave-one-out sensitivity analysis to evaluate the influence of each SNP on the outcome. To assess heterogeneity, we calculated the Cochrane’s Q value. To detect potential horizontal pleiotropy, we employed the MR-Egger intercept method. When horizontal pleiotropy was identified, we removed the outliers and applied the IVW method to combine the effect sizes of each SNP. This comprehensive approach ensured the robustness of our findings and minimized the risk of bias in our MR analysis.

Multivariable MR (MVMR) is an extension of MR that allows estimation of the causal effects of multiple exposures on outcomes [29]. We used MVMR to analyse multiple exposures. The analysis employed an IVW approach, which incorporated different phenotypes into MR analysis as a single exposure.

### Annotation of the GWAS results

We used the functional mapping and annotation (FUMA) software to pinpoint independent significant SNPs (IndSigSNPs) and map them to corresponding genes while identifying genomic regions free from LD [30]. Specifically, we mapped all genes situated within 10 kb of each variant. IndSigSNPs were extracted when their P-value satisfied genome-wide significance criteria (P ≤ 5.0E−08) and did not exhibit LD with each other (r2 < 0.6). To identify lead SNPs, we selected a subset of independent significant SNPs that demonstrated LD with each other at r2 < 0.1 within a 500 kb window. Subsequently, we determined genomic risk loci by merging lead SNPs located less than 500 kb from one another. Clumping procedures were executed using the European 1000 Genomes Project phase 3 reference panel. These methodologies allowed us to thoroughly map and annotate SNPs and to identify pertinent genomic risk loci associated with the phenotype under investigation.

### Cross-trait meta-analysis of COPD and CRC

We carried out a cross-trait meta-analysis to uncover pleiotropic genetic variants shared between COPD and CRC [31]. ASSET, an unbiased approach, facilitates cross-trait meta-analysis by permitting a subset of input GWASs to exhibit no effect on a specific SNP. It developed a multi-test adjustment program capable of efficiently accounting for the correlation among different test statistics. This method identifies the most robust association signal by exhaustively exploring all possible subsets of GWASs and their inputs within a fixed-effect framework. Significant genes were subjected to gene network analysis using the STRING database (https://string-db.org/), and the resulting gene interaction network was subsequently imported into Cytoscape (version 3.7.2) for protein–protein interaction (PPI) visualization.

### Weighted gene co-expression network analysis (WCGNA) of CRC and COPD

WGCNA is a system biology approach that can identify co-expression modules of genes and explore their associations with biological features or diseases. We have selected 0.85 as the soft-thresholding value, which ensures that our network structure aligns with the characteristics of a scale-free network. We have employed the dynamic tree cut method to identify different modules, aiming to investigate if any module correlates with fatty acids [32]. By constructing a co-expression network, WGCNA clusters genes with similar expression patterns and identifies modules most relevant to the clinical phenotype of diseases [33]. In this study, WGCNA package (version 1.72) was used to cluster and identify genes into different modules in four datasets, GSE15781, GSE29621, GSE17536, and GSE57148. Genes from modules were extracted for KEGG enrichment analysis.

### Gene enrichment analysis

We extracted all pathways of *Homo sapiens* from KEGG as the background gene set for enrichment analysis and performed enrichment analysis on genes from different modules separately in four datasets [34]. The modules enriched in lipid-related pathways were used for Pearson correlation analysis to investigate the correlation between the modules of the COPD dataset and the modules of the CRC dataset containing fatty acid pathways.

### Statistics

For NHANES, to address population representativeness, we used recommended 2-year sampling weights from NHANES 2003–2004. The analyses for NHANES were weighted using the sampling weights provided for each dataset so that the results are representative of the national US population [35]. Comparisons were analyzed with the weighted t -test for normally distributed data, or the weighted Wilcoxon signed-rank test for nonnormal data. We also used weighted logistic regression analysis to determine the relative risk of colon cancer occurrence in relation to the characteristics of NHANES participants. Dose–response relations were examined by using weighted restricted cubic spline analysis. Statistical analysis was performed using R software (version 4.2.2). A P-value < 0.05 was considered statistically significant.

### Role of funders

Our study utilized data obtained exclusively from publicly available databases and received no substantial funding from any specific source.

## Results

### Study design

We conducted a search on Pubmed for articles relating to both COPD and CRC, and we identified a total of 217 pieces. The majority of these articles imply that COPD serves as one of the risk factors for CRC, yet none expounds on the intermediary role of fatty acids between the two conditions.

**Fig. 1:**
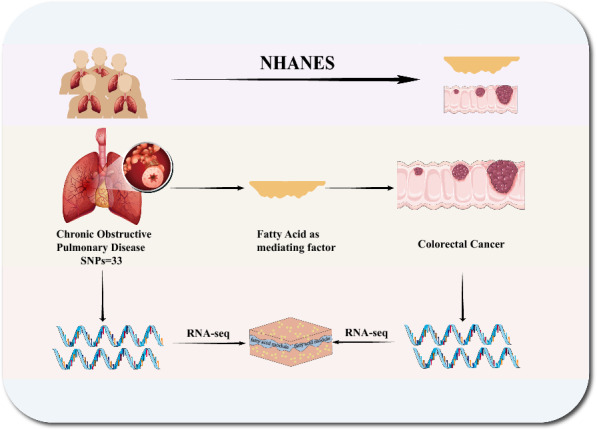
Exploring multi-level investigation using NHANES, GWAS, and GEO databases

To explore the association of risk of CRC development or progression with COPD via the fatty acid pathway, we performed an initial investigation using clinical databases, followed by a validation study using genomic and transcriptomic data. A schematic representation of the study is shown in Fig. 1. All data sources used in the study are listed in Additional file 1: Table S1.

### Omega-3 fatty acids and COPD are risk factors for CRC

This study included 1729 NHANES participants, representing 179.2 million non-institutionalized US residents. Of the total study population, 11 participants (1%) had colon cancer and 41 (2%) self-reported COPD. There was a trend towards higher levels of omega-3 fatty acids and lower levels of the omega-6/omega-3 ratio in the COPD population compared with levels in the non-COPD population (Table 1). Similar results were obtained in the colon cancer subgroups.

**Table 1:** Characteristics of participants in NHANES

Characteristics	According to COPD			According to colon cancer		
	Non COPD (N = 1688)	COPD (N = 41)	P	Non colon cancer (N = 1718)	Colon cancer (N = 11)	P
Overall Omega-3 fatty acids (µmol/l)	235.4 (179.4, 311.5)	240.6 (167.0, 344.2)	0.87	235.4 (178.7, 311.2)	330.7 (202.8, 421)	0.35
Alpha-linolenic acid (µmol/l)	61.5 (46.3, 85.3)	59.7 (39.7, 73.6)	0.34	61.2 (46.2, 84.7)	60.9 (41.2, 102)	0.72
Eicosapentaenoic acid (µmol/l)	41 (29.1, 60.2)	50.5 (33.0, 63.7)	0.18	41.0 (29.2, 60.4)	57.8 (35.6, 68.3)	0.21
Docosahexaenoic acid (µmol/l)	121 (91.6, 168)	124 (84.1, 167)	0.96	121 (91.4, 168)	183 (124, 212)	0.24
Overall Omega-6 fatty acids (µmol/l)	4252 (3739, 4860)	3997 (3456, 4750)	0.05	4246 (3731, 4850)	4232 (3754, 5460)	0.92
Linoleic acid (µmol/l)	3450 (2980, 3980)	3230 (2710, 3660)	0.004	3450 (2970, 3970)	3340 (2860, 3990)	0.55
Arachidonic acid (µmol/l)	791 (650, 929)	813 (678, 924)	0.87	791 (650, 928)	921 (624, 1050)	0.38
Ratio Omega-6/Omega-3	18.83 (0.36)	16.94 (1.82)	0.25	18.4 (14.8, 22.5)	15.1 (11.4, 20.9)	0.15

*COPD* chronic obstructive pulmonary disease

P value < 0.05 was considered statistically significant

**Fig. 2:**
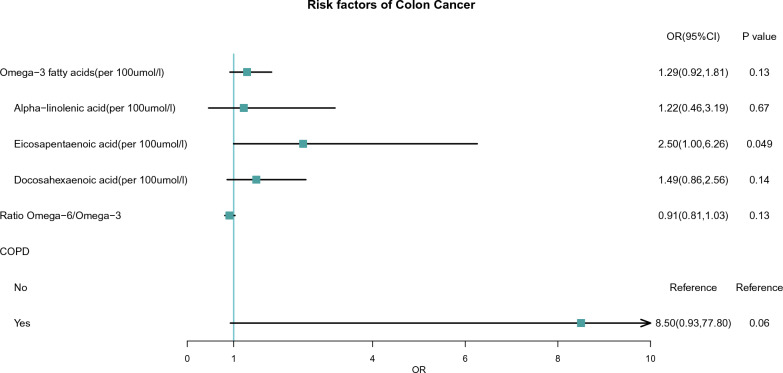
Effects of characteristics on risk of colon cancer in NHANES. P value < 0.05 was considered statistically significant. *OR* odds ratio, *CI* confidence intervals, *COPD* chronic obstructive pulmonary disease

Using a weighted logistic regression analysis, we analysed the effects of COPD, omega-3 fatty acids and omega-6/omega-3 on the risk of colon cancer. We found a corresponding increase in the risk of colon cancer in the group with increased levels of omega-3 fatty acids and COPD, though this was not statistically significant. The effect of eicosapentaenoic acid was statistically significant (p < 0.05) (Fig. 2). In contrast, elevated levels of the omega-6/omega-3 ratio appeared to play a protective role in the development of colon cancer. The effect of omega-3 fatty acids and omega-6/omega-3 ratio on colon cancer was verified in a dose–response curve. The results revealed a linear relationship between these factors and the risk of colon cancer (NL-P-value > 0.05) (Additional file 1: Fig. S2A, B).

### Assessment of the causal relationship between COPD and CRC using MR analysis

**Fig. 3:**
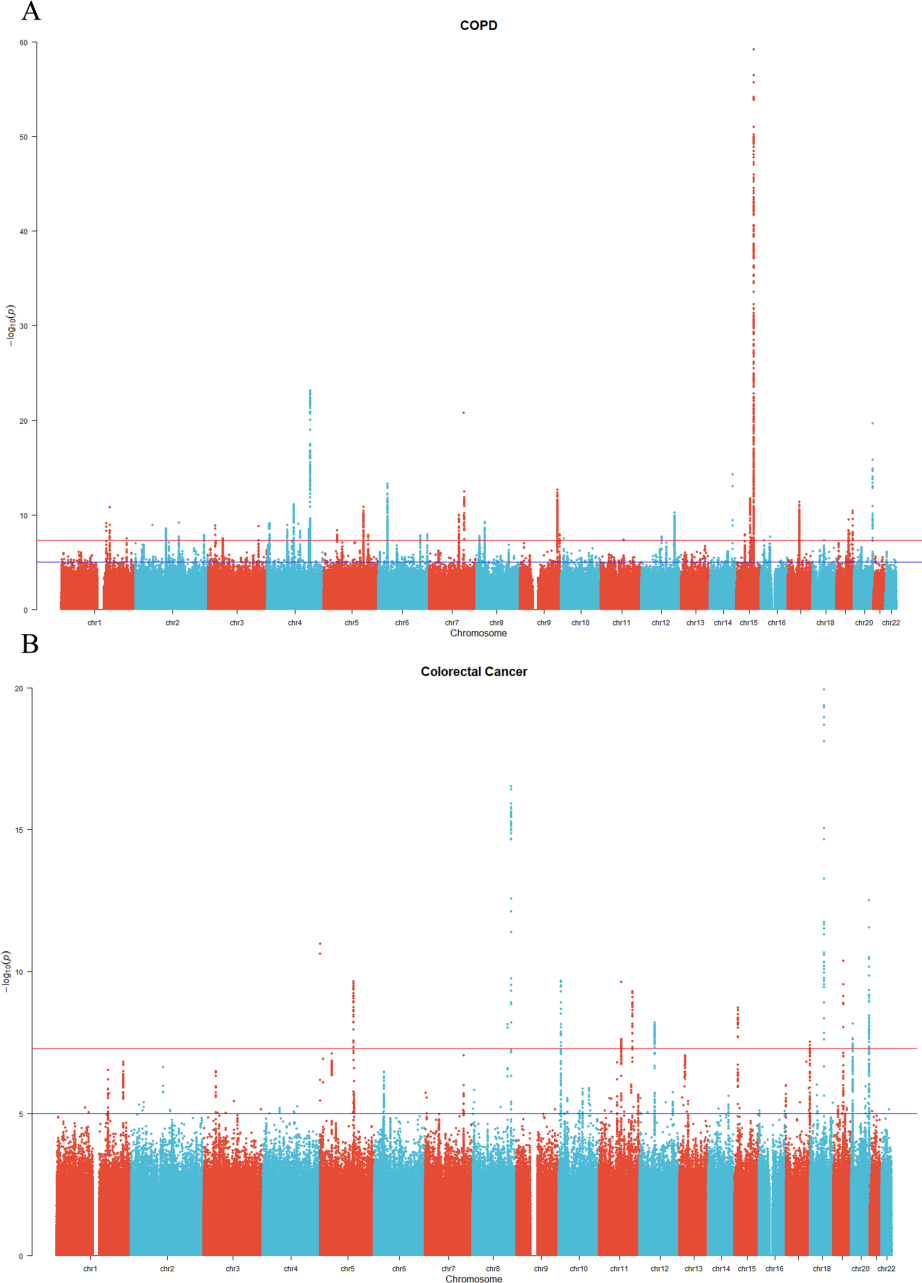
Manhattan plot of GWAS results of the COPD outcomes and Colorectal Cancer. The x-axis represents the SNPs locus on the chromosome, and the y-axis represents the p-value of the SNPs locus. The red and blue reference lines represent −log10 logarithm of p-values of 5e−08 and 1e−05, respectively. *COPD* chronic obstructive pulmonary disease

We performed a computation of the R^2^ and F statistics for the loci where the p-values were less than 5e−8 in the GWAS data of COPD (Additional file 1: Tables S2, S3). As shown in Table 2 and the scatter plot in Additional file 1: Fig. S3, the IVW results indicated a positive genetic correlation between COPD and CRC (OR and 95% CI 1.17, 1.01–1.36; p = 0.036). The MR-Egger regression analysis did not confirm the presence of directional pleiotropy for the genetic instrumental variables in either of the causal relationships (P > 0.05). The results of the leave-one-out analysis indicated that certain rsID positions exceeded the invalid vertical lines; however, this occurrence does not adversely impact our causal inference (Additional file 1: Fig. S4). The Manhattan plot was used to display the relationship between all loci and their corresponding p-values for the 22 chromosomes; the red reference line represents the p-value threshold of 5e−08 (Fig. 3A, B).

**Table 2:** Causal effects of COPD on colorectal cancer outcome

Exposure	Outcome	Method	nSNP	OR [95% CI]	P
COPD	Colorectal cancer	MR Egger	33	1.34 [0.88–2.04]	0.177
COPD	Colorectal cancer	Weighted median	33	1.13 [0.89–1.43]	0.322
COPD	Colorectal cancer	IVW	33	1.17 [1.01–1.36]	0.036
COPD	Colorectal cancer	Simple mode	33	1.07 [0.70–1.62]	0.767
COPD	Colorectal cancer	Weighted mode	33	1.11 [1.50–1.81]	0.526

*IVW* inverse variance weighted, *COPD* chronic obstructive pulmonary disease

### Fatty acids were identified as an optimal mediator by MVMR analysis

**Fig. 4:**
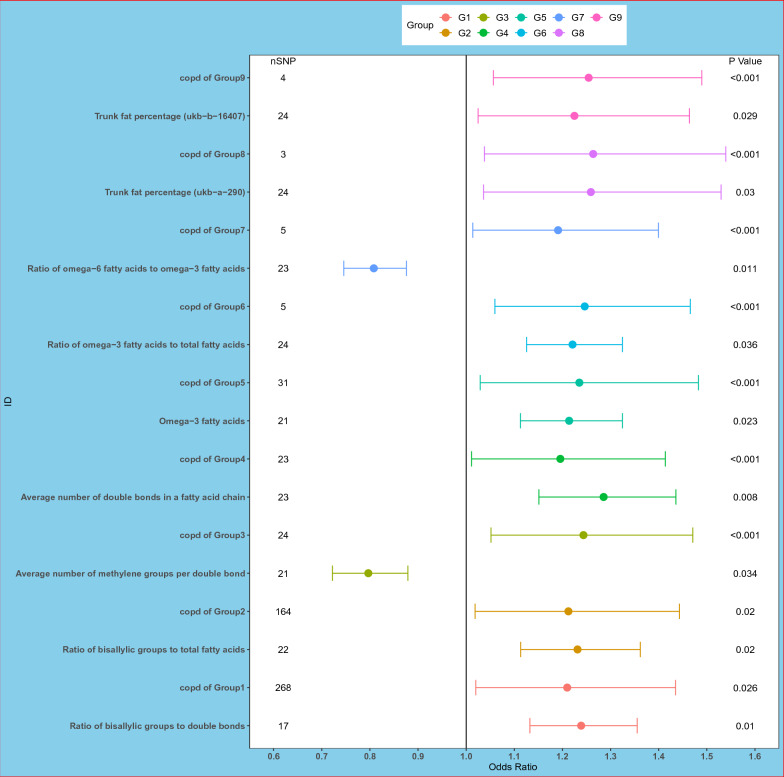
This forest plot represents the 9 lipid-related mediators identified by MVMR analysis with COPD as exposure and colorectal cancer as outcome. An odds ratio (OR) greater than 1 indicates that the corresponding mediator increases the risk of colorectal cancer, while an OR less than 1 indicates a protective effect. *MVMR* Multivariable Mendelian Randomization analysis

We used a two-sample MR approach, with the COPD GWAS dataset as the exposure and lipid-related GWAS data from the UK Biobank as the outcome, and retained results with statistically significant p-values. We also conducted a MR analysis using lipid-related data from the UK Biobank as the exposure and CRC GWAS dataset as the outcome. We then examined the intersection of these results with those of the previous analysis. A total of 28 candidate mediators were ultimately identified for MVMR analysis. MVMR analyses were conducted for the 28 exposures by combining them with COPD as the exposure and assessing their potential mediation effect on CRC. The results identified a total of nine lipid-related factors as potential mediators linking COPD and CRC. These mediators were significantly associated with omega-3 and omega-6 fatty acids and body fat percentage (Fig. 4). All lipid-related potential mediators used in MVMR analysis are shown in Additional file 1: Table S3.

### Cross-trait meta-analysis and annotation of COPD and CRC GWAS

**Fig. 5:**
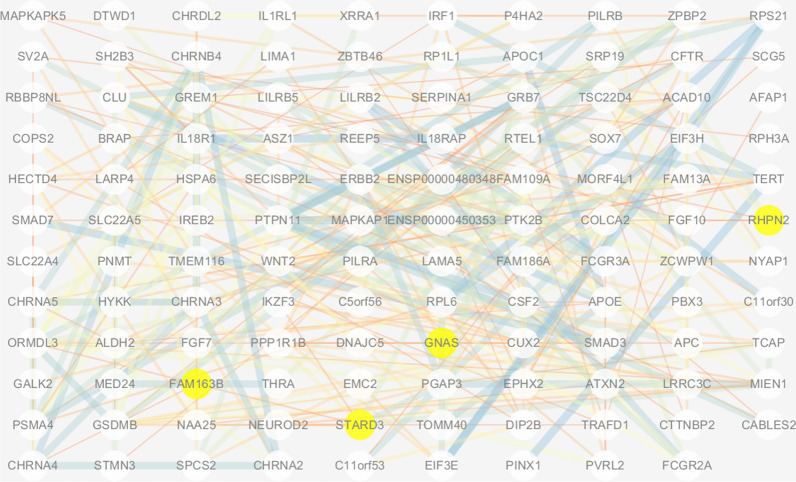
Protein–protein interaction network diagram annotated from genes obtained after performing cross-trait meta-analysis between COPD and CRC. The yellow nodes represent four genes (GNAS, RHPN2, FAM163B, STARD3), which were annotated with SNPs that have p-values smaller than 5e−08

Using a cross-phenotype meta-analysis, we identified a set of 43 loci that demonstrated statistically significant correlations with both COPD and CRC. Notably, four of these loci, specifically GNAS, FAM163B, RHPN2, and STARD3, were annotated as genes (Additional file 1: Table S4). We annotated loci that exhibited significant associations with any trait, resulting in a total of 156 genes (Additional file 1: Table S5). Among these genes, we obtained a gene interaction network from STRING and imported 119 genes into Cytoscape for analysis, ultimately generating a PPI network (Fig. 5).

Gene annotation was performed on loci with GWAS p-values less than 5e−08 for COPD and CRC; the results are shown in Additional file 1: Tables S6 and S7. A total of 274 and 79 genes were annotated for COPD and CRC GWAS, respectively.

### Identification of fatty acid module in RNA expression profiles of COPD and CRC

**Fig. 6:**
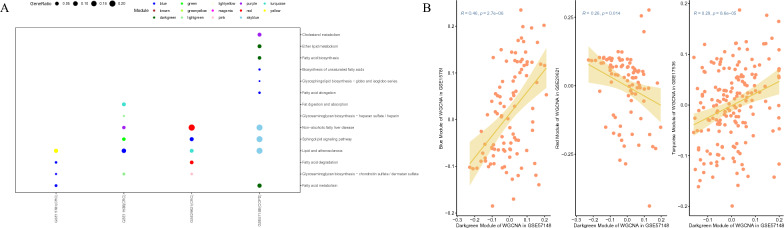
**A** Visualization of lipid-related modules enriched through WGCNA analysis across GSE29621, GSE17536, GSE15781, and GSE57148 datasets. The size of each dot represents the number of genes enriched in the corresponding pathway, while the color represents the respective module. **B** Extracting fatty-acid-related modules and conducting correlation analysis using module eigengenes in COPD and CRC

When the soft threshold power was above the reference line with a value of 0.85, the connectivity between genes in the gene network satisfied the scale-free network distribution. Co-expression modules were extracted for each dataset by applying a phylogenetic tree-based clustering algorithm (Additional file 1: Fig. S5A–D). We performed KEGG metabolic pathway enrichment analysis for genes within each module, and several modules in all four datasets were identified as significantly enriched in fatty acid metabolism, elongation, or degradation pathways (Fig. 6A).

### Correlation analysis of fatty acid modules between COPD and CRC

The module eigengene, which represents the expression pattern of gene modules, was used to analyse the correlation between fatty acid-enriched modules in COPD and CRC datasets. Specifically, we calculated the module eigengenes for the relevant modules in GSE29621, GSE17536, GSE15781, and GSE57148 and determined their intercorrelations. The blue module in GSE15781 and the turquoise module in GSE17536 were positively correlated with the dark green module in GSE57148. We observed a negative correlation between the red module in GSE29621 and the dark green module in GSE57184, which could be attributed to the enrichment of the red module in GSE29621 in fatty acid degradation pathways (Fig. 6B). The negative correlation between the red module in GSE29621 and the dark green module in GSE57184 could indicate a potentially harmful effect of fatty acid degradation on CRC development.

## Discussion

In the present study, we aimed to elucidate the intricate relationship between COPD, CRC, and fatty acid metabolism, with a focus on understanding the underlying mechanisms and potential causal connections. Our investigation has provided compelling evidence that COPD is a significant risk factor for CRC development and that these two conditions share a strong genetic correlation. A nationwide retrospective analysis revealed that regardless of smoking status, COPD contributes to the progression of CRC [36]. In another population-based cohort study conducted nationwide, COPD was associated with poorer survival outcomes [37]. This provides a solid foundation for further investigation into the association between fatty acids and the two diseases.

We propose that fatty acids serve as mediators connecting COPD and CRC and that imbalances in omega-3 and omega-6 fatty acids may lead to an increased risk of CRC in COPD patients. To validate the role of fatty acids as mediators between COPD and CRC, we used cutting-edge multi-omics approaches, incorporating bioinformatics analyses and the examination of clinical data from the NHANES database [38]. We suggest that omega-3 fatty acids exhibit anti-inflammatory properties in COPD, while omega-6 fatty acids promote inflammation, which has been corroborated by previous research [39–41]. The reduction in pulmonary inflammation leads to a noticeable downregulation of omega-6 fatty acids, thereby contributing to an overall protective effect in the lung. Notably, our study proposes that both omega-3 and omega-6 fatty acids possess pro-inflammatory characteristics in CRC. We observed an elevation in omega-3 levels, which appear to be detrimental in CRC patients, while a reduction in omega-6 levels was noted. This finding contradicts the majority of previous studies, which have posited that omega-3 fatty acids can be used for the treatment and reduction of mortality risk associated with CRC [42, 43]. The effects of Omega-3 and omega-6 fatty acids on CRC patients with COPD may be different compared with their effects on patients with CRC alone, and these effects may potentially be harmful. Nonetheless, some studies have corroborated our findings, positing that specific omega-3 fatty acids, including EPA and DPA, could potentially elevate the risk of CRC [44]. We hypothesize that alterations in fatty acid composition among COPD patients may influence CRC development through the gut-lung axis crosstalk, leading to corresponding changes in intestinal lipid metabolism. Previous research has reported that the gut-lung crosstalk can result in intestinal dysfunction in COPD patients [45]. Furthermore, the omega-3 fatty acids induced by the gut microbiota can interfere with pulmonary lipid metabolism [46]. Whether the observed abnormalities in fatty acid metabolism in CRC patients with COPD are still associated with gut microbiota requires further investigation. Nevertheless, these findings provide reasonable evidence for the role of unsaturated fatty acids in influencing both COPD and CRC through the gut-lung axis.

Our study also identified four genes (GNAS, RHPN2, FAM163B, and STARD3 genes) with significant associations with both COPD and CRC through a rigorous cross-trait meta-analysis. GNAS has been associated with plasma free fatty acid and glycerol concentrations, participating in the cAMP signalling pathway to promote lipid breakdown [47, 48]. STARD3 promotes cholesterol accumulation within the organism [49]. Some studies have suggested that STARD3 may be a promising target for cancer treatment [50]. No lipid-related studies on RHPN2 and FAM163B have been reported. Investigating the relationship between these genes and fatty acid metabolism may facilitate the development of targeted therapeutic strategies or personalized dietary interventions, potentially achieving preventive or therapeutic benefits for patients with COPD and CRC.

This study possesses several key strengths. Firstly, the utilization of Mendelian randomization allows us to leverage genetic data as a bridge to probe the causal association between COPD and CRC. This approach mitigates the risk of reverse causation and minimizes potential confounding influences. Secondly, our research preliminarily demonstrates that Omega-3 and Omega-6 fatty acids can function as mediating factors between COPD and CRC. The multivariable MR analysis presented in Fig. 4 indicates that fatty acids can still function as mediating factors influencing colorectal cancer, even after adjustment for COPD. This underscores that our findings can aid researchers in gaining a deeper understanding of the potential shared mechanisms between these two diseases, providing a theoretical foundation for treatment. Lastly, our analyses consolidate findings from both genomic and transcriptomic data to substantiate our conclusions. The integration of phenomics with genomics and transcriptomics facilitates an understanding of the relationships between phenotypes and functional genes [51]. Our work thus provides a valuable resource for researchers in the field of phenomics, enabling a more profound exploration of COPD and CRC by integrating our findings.

It's important to note that while our research does not revolutionize existing prevention or treatment strategies, it can potentially refine them. For instance, a growing number of people are resorting to weight loss surgery to mitigate the risk of colorectal cancer [52]. As this surgery impacts the balance of fatty acids, our findings might offer a theoretical underpinning for this preventive treatment strategy, suggesting precision weight loss could be achieved by adjusting the ratio of Omega-3 to Omega-6 fatty acids. Moreover, patients undergoing chemotherapy often experience a disruption in their body's fatty acid ratio due to cellular damage caused by the treatment. For patients suffering from COPD in tandem with colorectal cancer, we propose the development of magnetically targeted green nanoparticle drugs aimed at the mitochondria, which could adjust the internal fatty acid ratio [53]. This study has several limitations. One limitation is the potential for confounding factors or pleiotropy in MR studies and the reliance on self-reported data for certain variables. Because of the limited sample size in our exploration of the NHANES database, some of our results were not statistically significant and require validation in larger clinical cohorts. Additionally, further research is required to bolster the bioinformatics validation by incorporating high-quality RNA expression profiles. Our study population consisted of individuals from a Western population. Given that diet-induced inflammation may be one mechanism linking the Western diet to COPD, whether such a causal relationship is present in patients from other regions remains to be investigated [54].

## Conclusion

In conclusion, our study offers valuable insights into the relationship between COPD, CRC, and fatty acid metabolism, illuminating the potential causal connections and mediating factors. Further research is required to confirm our findings and explore their clinical implications. These findings may help guide advancements in the diagnosis and treatment of both COPD and CRC.

### Supplementary Information

**Additional file 1: Table S1.** Sources of all data used in the study. **Table S2.** Independent SNPs associated with COPD. **Table S3.** 28 significant intermediate factors identified through two-sample MR analysis for both CRC and COPD. **Table S4.** The results of cross-trait meta-analysis of COPD and CRC. **Table S5.** Annotating the loci obtained from cross-trait meta-analysis. **Table S6.** Annotation of genomic loci with a p-value less than 5e−08 in the context of COPD. **Table S7.** Annotation of genomic loci with a p-value less than 5e−08 in the context of CRC. **Figure S1.** Flow chart of study participant selection process in NHANES. **Figure S2.** Dose-response association among fatty acids with colon caner. **Figure S3.** Causal effects of COPD on CRC. **Figure S4.** Leave-one-out analysis for the association of COPD and CRC. **Figure S5.** Cluster Dendrogram of CRC and COPD RNA-seq datasets.

## Data Availability

GWAS data are available through the MRC IEU Open GWAS database (http://gwas.mrcieu.ac.uk/). NHANES data are publicly available through the Center for Disease Control. (https://wwwn.cdc.gov/nchs/nhanes/). The datasets generated and/or analysed during the current study are available in the GEO database (https://www.ncbi.nlm.nih.gov/geo/).

## Abbreviations

COPD: Chronic obstructive pulmonary disease
CRC: Colorectal cancer
WGCNA: Weighted gene co-expression network analysis
KEGG: Kyoto Encyclopedia of Genes and Genomes enrichment
MR: Mendelian randomization
NHANES: National health and nutrition examination survey
GEO: Gene expression omnibus
GWAS: Genome wide association study
IVW: Inverse-variance weighted
LD: Linkage disequilibrium
SNP: Single nucleotide polymorphism
CDC: Centers for Disease Control and Prevention
GLOBOCAN: New global cancer data
STRING: Search tool for the retrieval of interaction gene/proteins
PPI: Protein–protein interaction networks
EPA: Eicosapentaenoic acid
DPA: Docosahexaenoic acid
GNAS: Guanine nucleotide binding protein (G Protein), alpha stimulating activity polypeptide 1
RNPN2: Rhophilin-like Rho-GTPase binding protein
STARD3: Start domain-containing protein 3
FAM163B: Family with sequence similarity 163, member B
RNA: Ribonucleic acid
OR: Odds ratio
EUR: European
AFR: African
AMR: Admixed American
EAS: East Asian
MID: Middle Eastern
CSA: Central and South Asian
MVMR: Multivariable Mendelian randomization
IndSigSNPs: Independent significant SNPs
US: United States
UK: United Kingdom
cAMP: Cyclic adenosine monophosphate
GBMI: Global Biobank Meta-analysis Initiative
WGS: Whole-genome sequencing
GECCO: Genetic Epidemiology of Colorectal Cancer Consortium
CCFR: Colorectal Cancer Family Registry
CORECT: Colon Cancer Family Registry
FUMA: Functional mapping and annotation
