# Supplementary material for: Interplay of chronic obstructive pulmonary disease and colorectal cancer development: unravelling the mediating role of fatty acids through a comprehensive multi-omics analysis
Source: J Transl Med. 2023 Sep 1;21:587. doi: 10.1186/s12967-023-04278-1 (PMC10474711; doi:10.1186/s12967-023-04278-1)
Supplement: Supplementary file 1 — Additional file 1: Table S1. Sources of all data used in the study. Table S2. Independent SNPs associated with COPD. Table S3. 28 significant intermediate factors identified through two-sample MR analysis for both CRC and COPD. Table S4. The results of cross-trait meta-analysis of COPD and CRC. Table S5. Annotating the loci obtained from cross-trait meta-analysis. Table S6. Annotation of genomic loci with a p-value less than 5e−08 in the context of COPD. Table S7. Annotation of genomic loci with a p-value less than 5e−08 in the context of CRC. Figure S1. Flow chart of study participant selection process in NHANES. Figure S2. Dose-response association among fatty acids with colon caner. Figure S3. Causal effects of COPD on CRC. Figure S4. Leave-one-out analysis for the association of COPD and CRC. Figure S5. Cluster Dendrogram of CRC and COPD RNA-seq datasets. [file 12967_2023_4278_MOESM1_ESM.docx]

**Additional file 1**

Table S1 Sources of all data used in the study.. 2

Table S2 Independent SNPs associated with COPD. 3

Table S3 28 significant intermediate factors identified through two-sample MR analysis for both CRC and COPD. 5

Table S4 The results of cross-trait meta-analysis of COPD and CRC. 8

Table S5 Annotating the loci obtained from cross-trait meta-analysis. 10

Table S6 Annotation of genomic loci with a p-value less than 5e-08 in the context of COPD. 17

Table S7 Annotation of genomic loci with a p-value less than 5e-08 in the context of CRC. 28

Figure S1 Flow chart of study participant selection process in NHANES.. 32

Figure S2 Dose-response association among fatty acids with colon caner.. 33

Figure S3 Causal effects of COPD on CRC. 34

Figure S4 Leave-one-out analysis for the association of COPD and CRC. 35

Figure S5 Cluster Dendrogram of CRC and COPD RNA-seq datasets. 36

**Table S1** Sources of all data used in the study.

| **Consortia** | **Phenotype** | **Ancestry** | **Participants** | **Web Sources** |
| --- | --- | --- | --- | --- |
| NA | Colorectal Cancer | European | 64190 | <https://www.ebi.ac.uk/gwas/studies/GCST012879> |
| NA | COPD | European | 1392366 | <https://www.globalbiobankmeta.org/general-4> |
| NA | Ratio of bisallylic groups to double bonds | European | 13524 | <https://gwas.mrcieu.ac.uk/datasets/met-c-844/> |
| NA | Ratio of bisallylic groups to total fatty acids | European | 13171 | <https://gwas.mrcieu.ac.uk/datasets/met-c-845/> |
| NA | Average number of methylene groups per double bond | European | 13532 | <https://gwas.mrcieu.ac.uk/datasets/met-c-847/> |
| NA | Average number of double bonds in a fatty acid chain | European | 15728 | <https://gwas.mrcieu.ac.uk/datasets/met-c-851/> |
| NA | Omega-3 fatty acids | European | 114999 | <https://gwas.mrcieu.ac.uk/datasets/met-d-Omega_3/> |
| NA | Ratio of omega-3 fatty acids to total fatty acids | European | 114999 | https://gwas.mrcieu.ac.uk/datasets/met-d-Omega_3_pct/ |
| NA | Ratio of omega-6 fatty acids to omega-3 fatty acids | European | 114999 | <https://gwas.mrcieu.ac.uk/datasets/met-d-Omega_6_by_Omega_3/> |
| Neale Lab | Trunk fat percentage (ukb-a-290) | European | 331113 | <https://gwas.mrcieu.ac.uk/datasets/ukb-a-290/> |
| MRC-IEU | Trunk fat percentage (ukb-b-16407) | European | 454613 | <https://gwas.mrcieu.ac.uk/datasets/ukb-b-16407/> |
| NA | Colorectal Cancer | USA | 65 | https://www.ncbi.nlm.nih.gov/geo/query/acc.cgi?acc=GSE29621 |
| NA | Colorectal Cancer | Norway | 42 | https://www.ncbi.nlm.nih.gov/geo/query/acc.cgi?acc=GSE15781 |
| NA | Colorectal Cancer | USA | 177 | https://www.ncbi.nlm.nih.gov/geo/query/acc.cgi?acc=GSE17536 |
| NA | COPD | South Korea | 189 | https://www.ncbi.nlm.nih.gov/geo/query/acc.cgi?acc=GSE57148 |

COPD, chronic obstructive pulmonary disease; USA, United States of America

**Table S2** Independent SNPs associated with COPD.

| **SNP** | **OA** | **EA** | **BETA** | **SE** | **EAF** | **P** | **R^2^** | **F-statistic** |
| --- | --- | --- | --- | --- | --- | --- | --- | --- |
| rs11205303 | T | C | 0.037833 | 0.006155 | 0.3501 | 7.91E-10 | 2.76E-05 | 37.78204 |
| rs2099684 | A | G | 0.04219 | 0.006266 | 0.3045 | 1.67E-11 | 3.26E-05 | 45.33114 |
| rs4851569 | C | A | 0.035314 | 0.005946 | 0.3905 | 2.87E-09 | 2.53E-05 | 35.26839 |
| rs7598305 | G | T | 0.03863 | 0.006885 | 0.2383 | 2.02E-08 | 2.26E-05 | 31.47775 |
| rs62201158 | A | G | -0.06347 | 0.011168 | 0.07917 | 1.32E-08 | 2.32E-05 | 32.29675 |
| rs1286664 | C | T | -0.04107 | 0.00723 | 0.2134 | 1.35E-08 | 2.32E-05 | 32.26538 |
| rs1996664 | A | G | 0.051844 | 0.009362 | 0.1216 | 3.07E-08 | 2.33E-05 | 30.66487 |
| rs879394 | G | T | 0.040148 | 0.006647 | 0.2626 | 1.55E-09 | 2.62E-05 | 36.47743 |
| rs6839086 | G | T | -0.05835 | 0.009479 | 0.8263 | 7.44E-10 | 2.72E-05 | 37.90119 |
| rs13143549 | A | G | -0.03912 | 0.006429 | 0.4012 | 1.16E-09 | 2.70E-05 | 37.03392 |
| rs2464522 | G | A | 0.04505 | 0.006633 | 0.7112 | 1.11E-11 | 3.31E-05 | 46.12714 |
| rs77854845 | T | C | -0.05811 | 0.009938 | 0.1276 | 5.00E-09 | 3.22E-05 | 34.19017 |
| rs769672 | G | C | -0.03697 | 0.006251 | 0.316 | 3.33E-09 | 2.51E-05 | 34.97909 |
| rs13141641 | T | C | -0.06035 | 0.005993 | 0.4131 | 7.47E-24 | 7.28E-05 | 101.41 |
| rs10041294 | A | G | 0.033705 | 0.005923 | 0.4169 | 1.26E-08 | 2.33E-05 | 32.38758 |
| rs10077785 | C | T | 0.047867 | 0.007237 | 0.2058 | 3.73E-11 | 3.14E-05 | 43.75012 |
| rs11168049 | T | C | -0.03375 | 0.005945 | 0.4336 | 1.37E-08 | 2.31E-05 | 32.23121 |
| rs9272456 | T | A | 0.077124 | 0.010262 | 0.2665 | 5.66E-14 | 6.92E-05 | 56.48265 |
| rs7755011 | A | G | -0.03916 | 0.006977 | 0.4912 | 1.99E-08 | 2.39E-05 | 31.50071 |
| rs1322187 | G | T | 0.043645 | 0.007651 | 0.234 | 1.17E-08 | 2.57E-05 | 32.54283 |
| rs2405442 | T | C | -0.03868 | 0.006216 | 0.624 | 4.88E-10 | 2.78E-05 | 38.72691 |
| rs112534719 | T | C | 0.16909 | 0.023225 | 0.02287 | 3.33E-13 | 5.40E-05 | 53.00592 |
| rs11783247 | C | T | -0.03846 | 0.006926 | 0.4111 | 2.81E-08 | 2.29E-05 | 30.83533 |
| rs73229090 | C | A | -0.06515 | 0.0105 | 0.1077 | 5.49E-10 | 3.62E-05 | 38.49907 |
| rs12553980 | T | A | 0.045215 | 0.006357 | 0.6426 | 1.14E-12 | 3.83E-05 | 50.59431 |
| rs7130588 | A | G | 0.036268 | 0.00661 | 0.2655 | 4.08E-08 | 2.16E-05 | 30.10904 |
| rs12307208 | T | C | -0.06138 | 0.010962 | 0.09288 | 2.16E-08 | 2.77E-05 | 31.34958 |
| rs11525583 | G | A | 0.07145 | 0.011944 | 0.1798 | 2.20E-09 | 2.71E-05 | 35.78532 |
| rs112635299 | G | T | 0.18858 | 0.025296 | 0.01997 | 9.01E-14 | 5.87E-05 | 55.57603 |
| rs12232354 | C | T | -0.06534 | 0.01144 | 0.07365 | 1.12E-08 | 2.36E-05 | 32.61768 |
| rs10438355 | C | G | -0.04743 | 0.006755 | 0.2864 | 2.20E-12 | 3.56E-05 | 49.29536 |
| rs12908092 | T | C | 0.044191 | 0.007956 | 0.154 | 2.79E-08 | 2.23E-05 | 30.84853 |
| rs8040868 | T | C | 0.097373 | 0.005965 | 0.3761 | 6.60E-60 | 0.000193 | 266.4927 |
| rs4795400 | C | T | -0.04175 | 0.006121 | 0.4124 | 9.08E-12 | 3.46E-05 | 46.51778 |
| rs4803402 | G | A | 0.041859 | 0.007112 | 0.7356 | 3.97E-09 | 2.63E-05 | 34.6413 |
| rs383925 | T | C | -0.05121 | 0.007734 | 0.6514 | 3.54E-11 | 3.39E-05 | 43.85448 |
| rs6011779 | C | T | -0.06816 | 0.007366 | 0.8004 | 2.16E-20 | 6.15E-05 | 85.64107 |

SNP, single nucleotide polymorphism; EA, effect allele; OA, other allele; EAF, effect allele frequency; Beta, effect size; SE, standard error for beta; p, p-value; R^2^, phenotype variance explained by genetics.

**Table S3** 28 significant intermediate factors identified through two-sample MR analysis for both CRC and COPD.

| **Outcome** | **Exposure1** | **nSNP** | **beta** | **se** | **up95** | **low95** | **P** | **Exposure2** | **nSNP** | **beta** | **se** | **up95** | **low95** | **P** |
| --- | --- | --- | --- | --- | --- | --- | --- | --- | --- | --- | --- | --- | --- | --- |
| Colorectal Cancer | COPD | 24 | 0.19 | 0.087 | 0.36 | 0.02 | 0.029 | Ratio of bisallylic groups to double bonds \|\| id:met-c-844 | 4 | 0.21 | 0.046 | 0.30 | 0.12 | <0.01 |
| Colorectal Cancer | COPD | 24 | 0.19 | 0.089 | 0.37 | 0.02 | 0.030 | Ratio of bisallylic groups to total fatty acids \|\| id:met-c-845 | 3 | 0.21 | 0.051 | 0.31 | 0.11 | <0.01 |
| Colorectal Cancer | COPD | 23 | 0.22 | 0.086 | 0.39 | 0.05 | 0.011 | Average number of methylene groups per double bond \|\| id:met-c-847 | 5 | -0.23 | 0.050 | -0.13 | -0.33 | <0.01 |
| Colorectal Cancer | COPD | 24 | 0.18 | 0.085 | 0.35 | 0.01 | 0.036 | Average number of double bonds in a fatty acid chain \|\| id:met-c-851 | 5 | 0.25 | 0.056 | 0.36 | 0.14 | <0.01 |
| Colorectal Cancer | COPD | 22 | 0.22 | 0.09 | 0.39 | 0.05 | 0.01 | Phospholipids in large LDL \|\| id:met-c-885 | 13 | 0.07 | 0.04 | 0.15 | -0.01 | 0.09 |
| Colorectal Cancer | COPD | 21 | 0.21 | 0.093 | 0.39 | 0.03 | 0.023 | Omega-3 fatty acids \|\| id:met-d-Omega_3 | 31 | 0.19 | 0.044 | 0.28 | 0.11 | <0.01 |
| Colorectal Cancer | COPD | 23 | 0.22 | 0.083 | 0.38 | 0.06 | 0.008 | Ratio of omega-3 fatty acids to total fatty acids \|\| id:met-d-Omega_3_pct | 23 | 0.20 | 0.042 | 0.28 | 0.12 | <0.01 |
| Colorectal Cancer | COPD | 21 | 0.17 | 0.082 | 0.34 | 0.01 | 0.034 | Ratio of omega-6 fatty acids to omega-3 fatty acids \|\| id:met-d-Omega_6_by_Omega_3 | 24 | -0.21 | 0.041 | -0.13 | -0.29 | <0.01 |
| Colorectal Cancer | COPD | 21 | 0.17 | 0.09 | 0.35 | -0.01 | 0.06 | Body fat percentage \|\| id:ukb-a-264 | 178 | 0.26 | 0.12 | 0.50 | 0.03 | 0.03 |
| Colorectal Cancer | COPD | 16 | 0.27 | 0.10 | 0.47 | 0.08 | 0.01 | Leg fat mass (right) \|\| id:ukb-a-275 | 196 | 0.08 | 0.11 | 0.31 | -0.14 | 0.46 |
| Colorectal Cancer | COPD | 19 | 0.23 | 0.09 | 0.40 | 0.05 | 0.01 | Leg fat percentage (left) \|\| id:ukb-a-278 | 174 | 0.21 | 0.15 | 0.50 | -0.09 | 0.17 |
| Colorectal Cancer | COPD | 13 | 0.27 | 0.10 | 0.47 | 0.07 | 0.01 | Arm fat percentage (right) \|\| id:ukb-a-282 | 156 | 0.18 | 0.13 | 0.43 | -0.08 | 0.17 |
| Colorectal Cancer | COPD | 11 | 0.33 | 0.10 | 0.53 | 0.13 | <0.01 | Arm fat mass (right) \|\| id:ukb-a-283 | 179 | 0.06 | 0.09 | 0.24 | -0.12 | 0.54 |
| Colorectal Cancer | COPD | 12 | 0.30 | 0.10 | 0.50 | 0.10 | <0.01 | Arm fat percentage (left) \|\| id:ukb-a-286 | 167 | 0.08 | 0.13 | 0.33 | -0.18 | 0.55 |
| Colorectal Cancer | COPD | 12 | 0.30 | 0.10 | 0.50 | 0.10 | <0.01 | Arm fat mass (left) \|\| id:ukb-a-287 | 179 | 0.08 | 0.09 | 0.26 | -0.11 | 0.41 |
| Colorectal Cancer | COPD | 19 | 0.19 | 0.09 | 0.36 | 0.02 | 0.03 | Trunk fat percentage \|\| id:ukb-a-290 | 164 | 0.23 | 0.10 | 0.43 | 0.04 | 0.02 |
| Colorectal Cancer | COPD | 17 | 0.30 | 0.09 | 0.48 | 0.12 | <0.01 | Trunk fat mass \|\| id:ukb-a-291 | 190 | 0.05 | 0.08 | 0.21 | -0.11 | 0.58 |
| Colorectal Cancer | COPD | 19 | 0.20 | 0.09 | 0.38 | 0.02 | 0.03 | Arm fat percentage (right) \|\| id:ukb-b-12854 | 272 | 0.16 | 0.12 | 0.40 | -0.08 | 0.20 |
| Colorectal Cancer | COPD | 17 | 0.23 | 0.09 | 0.40 | 0.06 | 0.01 | Trunk fat percentage \|\| id:ukb-b-16407 | 268 | 0.20 | 0.09 | 0.38 | 0.02 | 0.03 |
| Colorectal Cancer | COPD | 11 | 0.24 | 0.10 | 0.44 | 0.05 | 0.01 | Leg fat mass (right) \|\| id:ukb-b-18096 | 277 | 0.13 | 0.11 | 0.35 | -0.09 | 0.26 |
| Colorectal Cancer | COPD | 15 | 0.25 | 0.09 | 0.43 | 0.08 | <0.01 | Leg fat percentage (left) \|\| id:ukb-b-18377 | 256 | 0.19 | 0.14 | 0.47 | -0.09 | 0.18 |
| Colorectal Cancer | COPD | 9 | 0.32 | 0.10 | 0.52 | 0.13 | <0.01 | Whole body fat mass \|\| id:ukb-b-19393 | 289 | 0.06 | 0.09 | 0.23 | -0.11 | 0.50 |
| Colorectal Cancer | COPD | 11 | 0.32 | 0.10 | 0.52 | 0.13 | <0.01 | Trunk fat mass \|\| id:ukb-b-20044 | 293 | 0.08 | 0.08 | 0.24 | -0.08 | 0.34 |
| Colorectal Cancer | COPD | 18 | 0.22 | 0.09 | 0.39 | 0.04 | 0.02 | Arm fat percentage (left) \|\| id:ukb-b-20188 | 265 | 0.18 | 0.12 | 0.41 | -0.06 | 0.14 |
| Colorectal Cancer | COPD | 15 | 0.23 | 0.09 | 0.40 | 0.05 | 0.01 | Leg fat percentage (right) \|\| id:ukb-b-20531 | 253 | 0.21 | 0.14 | 0.48 | -0.06 | 0.13 |
| Colorectal Cancer | COPD | 10 | 0.23 | 0.10 | 0.43 | 0.04 | 0.02 | Arm fat mass (right) \|\| id:ukb-b-6704 | 289 | 0.14 | 0.09 | 0.32 | -0.04 | 0.13 |
| Colorectal Cancer | COPD | 9 | 0.27 | 0.10 | 0.46 | 0.07 | 0.01 | Arm fat mass (left) \|\| id:ukb-b-8338 | 300 | 0.09 | 0.09 | 0.27 | -0.08 | 0.31 |
| Colorectal Cancer | COPD | 19 | 0.19 | 0.08 | 0.35 | 0.03 | 0.02 | Body fat percentage \|\| id:ukb-b-8909 | 272 | 0.20 | 0.11 | 0.41 | -0.01 | 0.06 |

**Table S4** The results of cross-trait meta-analysis of COPD and CRC.

| **SNP** | **Pvalue** | **Pvalue.1** | **Pvalue.2** | **OR.1** | **CI.low** | **CI.high** | **OR.2** | **CI.low.** | **CI.high** | **Pheno.1** | **Pheno.2** | **Symbol** |
| --- | --- | --- | --- | --- | --- | --- | --- | --- | --- | --- | --- | --- |
| rs10411210 | 2.93E-09 | 9.8E-05 | 1.26E-06 | 1.036 | 1.018 | 1.055 | 0.879 | 0.835 | 0.926 | COPD | CRC | NA |
| rs111596452 | 4.56E-09 | 3.7E-08 | 0.005283 | 1.064 | 1.041 | 1.087 | 0.921 | 0.869 | 0.976 | COPD | CRC | NA |
| rs112270518 | 4.6E-09 | 6.32E-08 | 0.003116 | 1.063 | 1.04 | 1.087 | 0.918 | 0.868 | 0.972 | COPD | CRC | NA |
| rs113067637 | 5.31E-09 | 3.66E-08 | 0.006256 | 1.074 | 1.047 | 1.101 | 0.918 | 0.864 | 0.976 | COPD | CRC | NA |
| rs113382419 | 1.67E-08 | 9.35E-08 | 0.008127 | 1.063 | 1.039 | 1.087 | 0.925 | 0.873 | 0.98 | COPD | CRC | NA |
| rs113864525 | 2.3E-09 | 3.93E-08 | 0.00243 | 1.064 | 1.041 | 1.088 | 0.916 | 0.866 | 0.969 | COPD | CRC | NA |
| rs11881367 | 1.52E-09 | 6.03E-05 | 1.03E-06 | 1.04 | 1.02 | 1.06 | 0.878 | 0.834 | 0.925 | COPD | CRC | NA |
| rs12459751 | 7.84E-09 | 0.000168 | 2.04E-06 | 1.035 | 1.017 | 1.054 | 0.883 | 0.838 | 0.929 | COPD | CRC | NA |
| rs148428140 | 3.55E-09 | 5.86E-08 | 0.002566 | 1.071 | 1.045 | 1.098 | 0.917 | 0.866 | 0.97 | COPD | CRC | NA |
| rs17841839 | 5.5E-09 | 0.000728 | 3.27E-07 | 1.04 | 1.016 | 1.063 | 0.86 | 0.811 | 0.911 | COPD | CRC | NA |
| rs185799410 | 1.33E-08 | 2.76E-05 | 2.16E-05 | 1.099 | 1.052 | 1.149 | 0.799 | 0.72 | 0.886 | COPD | CRC | NA |
| rs28363937 | 1.34E-09 | 0.000153 | 3.54E-07 | 1.036 | 1.017 | 1.054 | 0.876 | 0.832 | 0.922 | COPD | CRC | NA |
| rs28505093 | 2.25E-09 | 2.28E-05 | 4.1E-06 | 1.043 | 1.023 | 1.063 | 0.885 | 0.84 | 0.932 | COPD | CRC | NA |
| rs28570619 | 1.55E-11 | 0.000721 | 7.33E-10 | 1.04 | 1.016 | 1.063 | 0.801 | 0.746 | 0.86 | COPD | CRC | NA |
| rs28626308 | 5.6E-10 | 0.000223 | 9.81E-08 | 1.044 | 1.02 | 1.068 | 0.857 | 0.809 | 0.907 | COPD | CRC | NA |
| rs28840750 | 3.47E-13 | 0.000244 | 4.28E-11 | 1.043 | 1.02 | 1.067 | 0.797 | 0.745 | 0.853 | COPD | CRC | NA |
| rs3025316 | 2.5E-08 | 1E-07 | 0.011574 | 1.062 | 1.039 | 1.086 | 0.929 | 0.878 | 0.984 | COPD | CRC | NA |
| rs3025343 | 6.55E-10 | 1.14E-08 | 0.002263 | 1.065 | 1.042 | 1.088 | 0.916 | 0.866 | 0.969 | COPD | CRC | FAM163B |
| rs3025360 | 8.89E-10 | 1.56E-08 | 0.002276 | 1.064 | 1.042 | 1.087 | 0.916 | 0.866 | 0.969 | COPD | CRC | NA |
| rs4860797 | 2.17E-09 | 0.025426 | 3.53E-09 | 1.039 | 1.005 | 1.074 | 0.964 | 0.952 | 0.976 | CRC | COPD | NA |
| rs56057809 | 3.54E-09 | 5.82E-08 | 0.002573 | 1.063 | 1.04 | 1.087 | 0.917 | 0.866 | 0.97 | COPD | CRC | NA |
| rs60507951 | 4.55E-09 | 8.47E-05 | 2.3E-06 | 1.037 | 1.018 | 1.056 | 0.883 | 0.839 | 0.93 | COPD | CRC | NA |
| rs6510329 | 8.8E-09 | 0.004165 | 9.32E-08 | 1.056 | 1.017 | 1.096 | 0.821 | 0.764 | 0.883 | COPD | CRC | NA |
| rs7249860 | 1.92E-09 | 6.06E-05 | 1.31E-06 | 1.04 | 1.02 | 1.06 | 0.88 | 0.835 | 0.926 | COPD | CRC | NA |
| rs7250288 | 2.55E-08 | 0.000187 | 6.31E-06 | 1.046 | 1.021 | 1.07 | 0.884 | 0.838 | 0.932 | COPD | CRC | NA |
| rs7255601 | 8.87E-10 | 5.82E-05 | 6.07E-07 | 1.04 | 1.02 | 1.06 | 0.876 | 0.832 | 0.923 | COPD | CRC | RHPN2 |
| rs7256899 | 9.49E-10 | 1.62E-05 | 2.34E-06 | 1.043 | 1.023 | 1.064 | 0.882 | 0.838 | 0.929 | COPD | CRC | NA |
| rs7258173 | 3E-09 | 7.98E-05 | 1.58E-06 | 1.039 | 1.02 | 1.059 | 0.881 | 0.836 | 0.928 | COPD | CRC | NA |
| rs73039426 | 1.92E-11 | 0.000502 | 1.32E-09 | 1.042 | 1.018 | 1.066 | 0.826 | 0.776 | 0.878 | COPD | CRC | NA |
| rs73039428 | 2.2E-11 | 0.000529 | 1.44E-09 | 1.041 | 1.018 | 1.066 | 0.826 | 0.776 | 0.879 | COPD | CRC | NA |
| rs73039431 | 6.93E-11 | 0.000276 | 9.08E-09 | 1.043 | 1.02 | 1.067 | 0.843 | 0.795 | 0.894 | COPD | CRC | NA |
| rs73039433 | 3.13E-11 | 1.46E-05 | 7.54E-08 | 1.05 | 1.027 | 1.074 | 0.85 | 0.802 | 0.902 | COPD | CRC | NA |
| rs73039434 | 6.66E-10 | 0.000132 | 1.99E-07 | 1.041 | 1.02 | 1.062 | 0.861 | 0.814 | 0.911 | COPD | CRC | NA |
| rs73149487 | 2.29E-08 | 0.000628 | 1.68E-06 | 1.173 | 1.07 | 1.285 | 0.925 | 0.896 | 0.955 | CRC | COPD | STMN3 |
| rs73585909 | 2.3E-09 | 5.17E-05 | 1.85E-06 | 1.038 | 1.019 | 1.057 | 0.882 | 0.838 | 0.929 | COPD | CRC | NA |
| rs73585910 | 2.47E-09 | 4.98E-05 | 2.07E-06 | 1.038 | 1.019 | 1.057 | 0.883 | 0.839 | 0.929 | COPD | CRC | NA |
| rs739447 | 2.23E-09 | 4.57E-08 | 0.002023 | 1.063 | 1.04 | 1.087 | 0.915 | 0.865 | 0.968 | COPD | CRC | NA |
| rs75596189 | 2.41E-08 | 2.33E-07 | 0.004772 | 1.06 | 1.037 | 1.084 | 0.921 | 0.869 | 0.975 | COPD | CRC | NA |
| rs76602912 | 7.6E-10 | 7.29E-06 | 4.13E-06 | 1.118 | 1.065 | 1.174 | 0.777 | 0.698 | 0.865 | COPD | CRC | GNAS |
| rs8104911 | 2.47E-09 | 5.67E-05 | 1.82E-06 | 1.04 | 1.02 | 1.06 | 0.881 | 0.837 | 0.928 | COPD | CRC | NA |
| rs8109970 | 2.91E-09 | 5.26E-05 | 2.32E-06 | 1.04 | 1.021 | 1.06 | 0.883 | 0.838 | 0.93 | COPD | CRC | NA |
| rs8112217 | 5.36E-12 | 0.000641 | 2.75E-10 | 1.04 | 1.017 | 1.064 | 0.799 | 0.745 | 0.856 | COPD | CRC | NA |
| rs9271426 | 6.34E-09 | 6.23E-09 | 0.044202 | 1.042 | 1.027 | 1.056 | 0 | 0 | 0.37 | COPD | CRC | NA |

Cl, confidence interval

**Table S5** Annotating the loci obtained from cross-trait meta-analysis.

| **ENSG** | **SYMBOL** | **CHR** | **START** | **END** | **TYPE** |
| --- | --- | --- | --- | --- | --- |
| ENSG00000159164 | SV2A | 1 | 1.5E+08 | 1.5E+08 | protein_coding |
| ENSG00000143368 | SF3B4 | 1 | 1.5E+08 | 1.5E+08 | protein_coding |
| ENSG00000014914 | MTMR11 | 1 | 1.5E+08 | 1.5E+08 | protein_coding |
| ENSG00000163113 | OTUD7B | 1 | 1.5E+08 | 1.5E+08 | protein_coding |
| ENSG00000143226 | FCGR2A | 1 | 1.61E+08 | 1.61E+08 | protein_coding |
| ENSG00000173110 | HSPA6 | 1 | 1.61E+08 | 1.61E+08 | protein_coding |
| ENSG00000203747 | FCGR3A | 1 | 1.62E+08 | 1.62E+08 | protein_coding |
| ENSG00000115602 | IL1RL1 | 2 | 1.03E+08 | 1.03E+08 | protein_coding |
| ENSG00000115604 | IL18R1 | 2 | 1.03E+08 | 1.03E+08 | protein_coding |
| ENSG00000115607 | IL18RAP | 2 | 1.03E+08 | 1.03E+08 | protein_coding |
| ENSG00000180251 | SLC9A4 | 2 | 1.03E+08 | 1.03E+08 | protein_coding |
| ENSG00000196526 | AFAP1 | 4 | 7760441 | 7941653 | protein_coding |
| ENSG00000138640 | FAM13A | 4 | 89647106 | 90032549 | protein_coding |
| ENSG00000196782 | MAML3 | 4 | 1.41E+08 | 1.41E+08 | protein_coding |
| ENSG00000164362 | TERT | 5 | 1253262 | 1295184 | protein_coding |
| ENSG00000070193 | FGF10 | 5 | 44303646 | 44389808 | protein_coding |
| ENSG00000129595 | EPB41L4A | 5 | 1.11E+08 | 1.12E+08 | protein_coding |
| ENSG00000134982 | APC | 5 | 1.12E+08 | 1.12E+08 | protein_coding |
| ENSG00000258864 | CTC-554D6.1 | 5 | 1.12E+08 | 1.12E+08 | protein_coding |
| ENSG00000272869 | CTC-487M23.8 | 5 | 1.12E+08 | 1.12E+08 | protein_coding |
| ENSG00000153037 | SRP19 | 5 | 1.12E+08 | 1.12E+08 | protein_coding |
| ENSG00000129625 | REEP5 | 5 | 1.12E+08 | 1.12E+08 | protein_coding |
| ENSG00000212643 | ZRSR1 | 5 | 1.12E+08 | 1.12E+08 | protein_coding |
| ENSG00000171444 | MCC | 5 | 1.12E+08 | 1.13E+08 | protein_coding |
| ENSG00000047188 | YTHDC2 | 5 | 1.13E+08 | 1.13E+08 | protein_coding |
| ENSG00000164400 | CSF2 | 5 | 1.31E+08 | 1.31E+08 | protein_coding |
| ENSG00000072682 | P4HA2 | 5 | 1.32E+08 | 1.32E+08 | protein_coding |
| ENSG00000131435 | PDLIM4 | 5 | 1.32E+08 | 1.32E+08 | protein_coding |
| ENSG00000197208 | SLC22A4 | 5 | 1.32E+08 | 1.32E+08 | protein_coding |
| ENSG00000197375 | SLC22A5 | 5 | 1.32E+08 | 1.32E+08 | protein_coding |
| ENSG00000197536 | C5orf56 | 5 | 1.32E+08 | 1.32E+08 | protein_coding |
| ENSG00000125347 | IRF1 | 5 | 1.32E+08 | 1.32E+08 | protein_coding |
| ENSG00000164270 | HTR4 | 5 | 1.48E+08 | 1.48E+08 | protein_coding |
| ENSG00000121716 | PILRB | 7 | 99933737 | 99965356 | protein_coding |
| ENSG00000085514 | PILRA | 7 | 99965153 | 99997719 | protein_coding |
| ENSG00000078487 | ZCWPW1 | 7 | 99998449 | 1E+08 | protein_coding |
| ENSG00000166925 | TSC22D4 | 7 | 1E+08 | 1E+08 | protein_coding |
| ENSG00000166924 | NYAP1 | 7 | 1E+08 | 1E+08 | protein_coding |
| ENSG00000105989 | WNT2 | 7 | 1.17E+08 | 1.17E+08 | protein_coding |
| ENSG00000154438 | ASZ1 | 7 | 1.17E+08 | 1.17E+08 | protein_coding |
| ENSG00000001626 | CFTR | 7 | 1.17E+08 | 1.17E+08 | protein_coding |
| ENSG00000077063 | CTTNBP2 | 7 | 1.17E+08 | 1.18E+08 | protein_coding |
| ENSG00000183638 | RP1L1 | 8 | 10463859 | 10569697 | protein_coding |
| ENSG00000171056 | SOX7 | 8 | 10581278 | 10697357 | protein_coding |
| ENSG00000258724 | SOX7 | 8 | 10582909 | 10697357 | protein_coding |
| ENSG00000254093 | PINX1 | 8 | 10622473 | 10697394 | protein_coding |
| ENSG00000171044 | XKR6 | 8 | 10753555 | 11058875 | protein_coding |
| ENSG00000215346 | AF131215.5 | 8 | 10983980 | 10987745 | protein_coding |
| ENSG00000120899 | PTK2B | 8 | 27168999 | 27316903 | protein_coding |
| ENSG00000120903 | CHRNA2 | 8 | 27317279 | 27337400 | protein_coding |
| ENSG00000120915 | EPHX2 | 8 | 27348296 | 27403081 | protein_coding |
| ENSG00000120885 | CLU | 8 | 27454434 | 27472548 | protein_coding |
| ENSG00000104408 | EIF3E | 8 | 1.09E+08 | 1.09E+08 | protein_coding |
| ENSG00000104412 | EMC2 | 8 | 1.09E+08 | 1.09E+08 | protein_coding |
| ENSG00000147677 | EIF3H | 8 | 1.18E+08 | 1.18E+08 | protein_coding |
| ENSG00000212993 | POU5F1B | 8 | 1.28E+08 | 1.28E+08 | protein_coding |
| ENSG00000119487 | MAPKAP1 | 9 | 1.28E+08 | 1.28E+08 | protein_coding |
| ENSG00000167081 | PBX3 | 9 | 1.29E+08 | 1.29E+08 | protein_coding |
| ENSG00000196990 | FAM163B | 9 | 1.36E+08 | 1.36E+08 | protein_coding |
| ENSG00000054938 | CHRDL2 | 11 | 74407474 | 74442430 | protein_coding |
| ENSG00000166435 | XRRA1 | 11 | 74518784 | 74660245 | protein_coding |
| ENSG00000118363 | SPCS2 | 11 | 74660292 | 74690076 | protein_coding |
| ENSG00000158636 | C11orf30 | 11 | 76155967 | 76264069 | protein_coding |
| ENSG00000150750 | C11orf53 | 11 | 1.11E+08 | 1.11E+08 | protein_coding |
| ENSG00000196167 | COLCA1 | 11 | 1.11E+08 | 1.11E+08 | protein_coding |
| ENSG00000214290 | COLCA2 | 11 | 1.11E+08 | 1.11E+08 | protein_coding |
| ENSG00000050405 | LIMA1 | 12 | 50569571 | 50677329 | protein_coding |
| ENSG00000269529 | AC140061.12 | 12 | 50690489 | 50691718 | protein_coding |
| ENSG00000185958 | FAM186A | 12 | 50720013 | 50790405 | protein_coding |
| ENSG00000161813 | LARP4 | 12 | 50786166 | 50873787 | protein_coding |
| ENSG00000066084 | DIP2B | 12 | 50898768 | 51142450 | protein_coding |
| ENSG00000123268 | ATF1 | 12 | 51157493 | 51214905 | protein_coding |
| ENSG00000111249 | CUX2 | 12 | 1.11E+08 | 1.12E+08 | protein_coding |
| ENSG00000198324 | FAM109A | 12 | 1.12E+08 | 1.12E+08 | protein_coding |
| ENSG00000111252 | SH2B3 | 12 | 1.12E+08 | 1.12E+08 | protein_coding |
| ENSG00000204842 | ATXN2 | 12 | 1.12E+08 | 1.12E+08 | protein_coding |
| ENSG00000089234 | BRAP | 12 | 1.12E+08 | 1.12E+08 | protein_coding |
| ENSG00000111271 | ACAD10 | 12 | 1.12E+08 | 1.12E+08 | protein_coding |
| ENSG00000257767 | RP11-162P23.2 | 12 | 1.12E+08 | 1.12E+08 | protein_coding |
| ENSG00000111275 | ALDH2 | 12 | 1.12E+08 | 1.12E+08 | protein_coding |
| ENSG00000089022 | MAPKAPK5 | 12 | 1.12E+08 | 1.12E+08 | protein_coding |
| ENSG00000198270 | TMEM116 | 12 | 1.12E+08 | 1.12E+08 | protein_coding |
| ENSG00000089248 | ERP29 | 12 | 1.12E+08 | 1.12E+08 | protein_coding |
| ENSG00000111300 | NAA25 | 12 | 1.12E+08 | 1.13E+08 | protein_coding |
| ENSG00000135148 | TRAFD1 | 12 | 1.13E+08 | 1.13E+08 | protein_coding |
| ENSG00000173064 | HECTD4 | 12 | 1.13E+08 | 1.13E+08 | protein_coding |
| ENSG00000089009 | RPL6 | 12 | 1.13E+08 | 1.13E+08 | protein_coding |
| ENSG00000179295 | PTPN11 | 12 | 1.13E+08 | 1.13E+08 | protein_coding |
| ENSG00000089169 | RPH3A | 12 | 1.13E+08 | 1.13E+08 | protein_coding |
| ENSG00000119698 | PPP4R4 | 14 | 94612465 | 94746072 | protein_coding |
| ENSG00000140093 | SERPINA10 | 14 | 94749650 | 94759608 | protein_coding |
| ENSG00000197249 | SERPINA1 | 14 | 94843084 | 94857030 | protein_coding |
| ENSG00000166922 | SCG5 | 15 | 32933877 | 32989299 | protein_coding |
| ENSG00000166923 | GREM1 | 15 | 33010175 | 33026870 | protein_coding |
| ENSG00000138593 | SECISBP2L | 15 | 49280673 | 49338760 | protein_coding |
| ENSG00000166200 | COPS2 | 15 | 49398268 | 49447858 | protein_coding |
| ENSG00000156958 | GALK2 | 15 | 49447853 | 49660066 | protein_coding |
| ENSG00000166262 | FAM227B | 15 | 49619159 | 49913128 | protein_coding |
| ENSG00000140285 | FGF7 | 15 | 49715293 | 49780972 | protein_coding |
| ENSG00000104047 | DTWD1 | 15 | 49913177 | 49937333 | protein_coding |
| ENSG00000166949 | SMAD3 | 15 | 67356101 | 67487533 | protein_coding |
| ENSG00000103591 | AAGAB | 15 | 67493371 | 67547533 | protein_coding |
| ENSG00000103599 | IQCH | 15 | 67547138 | 67794598 | protein_coding |
| ENSG00000136381 | IREB2 | 15 | 78729773 | 78793798 | protein_coding |
| ENSG00000188266 | HYKK | 15 | 78799906 | 78829714 | protein_coding |
| ENSG00000268838 | AC027228.1 | 15 | 78830023 | 78831288 | protein_coding |
| ENSG00000041357 | PSMA4 | 15 | 78832747 | 78841604 | protein_coding |
| ENSG00000169684 | CHRNA5 | 15 | 78857862 | 78887611 | protein_coding |
| ENSG00000080644 | CHRNA3 | 15 | 78885394 | 78913637 | protein_coding |
| ENSG00000117971 | CHRNB4 | 15 | 78916461 | 79020096 | protein_coding |
| ENSG00000136378 | ADAMTS7 | 15 | 79051545 | 79103773 | protein_coding |
| ENSG00000185787 | MORF4L1 | 15 | 79102829 | 79190475 | protein_coding |
| ENSG00000171532 | NEUROD2 | 17 | 37759789 | 37766030 | protein_coding |
| ENSG00000131771 | PPP1R1B | 17 | 37782993 | 37792879 | protein_coding |
| ENSG00000131748 | STARD3 | 17 | 37793318 | 37819737 | protein_coding |
| ENSG00000173991 | TCAP | 17 | 37820440 | 37822808 | protein_coding |
| ENSG00000141744 | PNMT | 17 | 37824234 | 37826728 | protein_coding |
| ENSG00000161395 | PGAP3 | 17 | 37827375 | 37853050 | protein_coding |
| ENSG00000141736 | ERBB2 | 17 | 37844167 | 37886679 | protein_coding |
| ENSG00000141741 | MIEN1 | 17 | 37884749 | 37887040 | protein_coding |
| ENSG00000141738 | GRB7 | 17 | 37894180 | 37903544 | protein_coding |
| ENSG00000161405 | IKZF3 | 17 | 37921198 | 38020441 | protein_coding |
| ENSG00000186075 | ZPBP2 | 17 | 38024417 | 38034149 | protein_coding |
| ENSG00000073605 | GSDMB | 17 | 38060848 | 38076107 | protein_coding |
| ENSG00000172057 | ORMDL3 | 17 | 38077294 | 38083854 | protein_coding |
| ENSG00000204913 | LRRC3C | 17 | 38097727 | 38101000 | protein_coding |
| ENSG00000008838 | MED24 | 17 | 38175350 | 38217468 | protein_coding |
| ENSG00000126351 | THRA | 17 | 38214543 | 38250120 | protein_coding |
| ENSG00000101665 | SMAD7 | 18 | 46446223 | 46477081 | protein_coding |
| ENSG00000131941 | RHPN2 | 19 | 33469499 | 33555794 | protein_coding |
| ENSG00000076650 | GPATCH1 | 19 | 33571786 | 33621448 | protein_coding |
| ENSG00000166359 | WDR88 | 19 | 33622996 | 33666701 | protein_coding |
| ENSG00000268797 | CTC-490E21.12 | 19 | 41307202 | 41404187 | protein_coding |
| ENSG00000130202 | PVRL2 | 19 | 45349432 | 45392485 | protein_coding |
| ENSG00000130204 | TOMM40 | 19 | 45393826 | 45406946 | protein_coding |
| ENSG00000130203 | APOE | 19 | 45409011 | 45412650 | protein_coding |
| ENSG00000130208 | APOC1 | 19 | 45417504 | 45422606 | protein_coding |
| ENSG00000105609 | LILRB5 | 19 | 54754263 | 54761164 | protein_coding |
| ENSG00000131042 | LILRB2 | 19 | 54777675 | 54785039 | protein_coding |
| ENSG00000170866 | LILRA3 | 19 | 54799854 | 54809952 | protein_coding |
| ENSG00000087460 | GNAS | 20 | 57414773 | 57486247 | protein_coding |
| ENSG00000130702 | LAMA5 | 20 | 60883011 | 60942368 | protein_coding |
| ENSG00000171858 | RPS21 | 20 | 60962172 | 60963576 | protein_coding |
| ENSG00000149679 | CABLES2 | 20 | 60963688 | 60982341 | protein_coding |
| ENSG00000130701 | RBBP8NL | 20 | 60985293 | 61002589 | protein_coding |
| ENSG00000101204 | CHRNA4 | 20 | 61975420 | 62009753 | protein_coding |
| ENSG00000197457 | STMN3 | 20 | 62271061 | 62284780 | protein_coding |
| ENSG00000258366 | RTEL1 | 20 | 62289163 | 62328416 | protein_coding |
| ENSG00000026036 | RTEL1-TNFRSF6B | 20 | 62290653 | 62330037 | protein_coding |
| ENSG00000130584 | ZBTB46 | 20 | 62375019 | 62462597 | protein_coding |
| ENSG00000269223 | AL158091.1 | 20 | 62474779 | 62475273 | protein_coding |
| ENSG00000183260 | ABHD16B | 20 | 62492566 | 62494341 | protein_coding |
| ENSG00000268858 | C20ORF135 | 20 | 62492879 | 62493217 | protein_coding |
| ENSG00000101150 | TPD52L2 | 20 | 62496596 | 62522898 | protein_coding |
| ENSG00000101152 | DNAJC5 | 20 | 62526518 | 62567384 | protein_coding |
| ENSG00000198276 | UCKL1 | 20 | 62571186 | 62587769 | protein_coding |

ENSG, ensembl gene; CHR, chromosome

**Table S6** Annotation of genomic loci with a p-value less than 5e-08 in the context of COPD.

| ENSG | SYMBOL | CHR | START | END | TYPE |
| --- | --- | --- | --- | --- | --- |
| ENSG00000159164 | SV2A | 1 | 1.5E+08 | 1.5E+08 | protein_coding |
| ENSG00000143368 | SF3B4 | 1 | 1.5E+08 | 1.5E+08 | protein_coding |
| ENSG00000014914 | MTMR11 | 1 | 1.5E+08 | 1.5E+08 | protein_coding |
| ENSG00000163113 | OTUD7B | 1 | 1.5E+08 | 1.5E+08 | protein_coding |
| ENSG00000143226 | FCGR2A | 1 | 1.61E+08 | 1.61E+08 | protein_coding |
| ENSG00000273112 | RP11-25K21.6 | 1 | 1.61E+08 | 1.62E+08 | processed_transcript |
| ENSG00000173110 | HSPA6 | 1 | 1.61E+08 | 1.61E+08 | protein_coding |
| ENSG00000224203 | RPS23P10 | 1 | 1.62E+08 | 1.62E+08 | pseudogene |
| ENSG00000203747 | FCGR3A | 1 | 1.62E+08 | 1.62E+08 | protein_coding |
| ENSG00000224515 | RP11-5K23.5 | 1 | 1.62E+08 | 1.62E+08 | antisense |
| ENSG00000115602 | IL1RL1 | 2 | 1.03E+08 | 1.03E+08 | protein_coding |
| ENSG00000115604 | IL18R1 | 2 | 1.03E+08 | 1.03E+08 | protein_coding |
| ENSG00000236785 | AC007248.7 | 2 | 1.03E+08 | 1.03E+08 | pseudogene |
| ENSG00000115607 | IL18RAP | 2 | 1.03E+08 | 1.03E+08 | protein_coding |
| ENSG00000264764 | MIR4772 | 2 | 1.03E+08 | 1.03E+08 | miRNA |
| ENSG00000236525 | AC007278.2 | 2 | 1.03E+08 | 1.03E+08 | sense_intronic |
| ENSG00000234389 | AC007278.3 | 2 | 1.03E+08 | 1.03E+08 | sense_intronic |
| ENSG00000180251 | SLC9A4 | 2 | 1.03E+08 | 1.03E+08 | protein_coding |
| ENSG00000226674 | TEX41 | 2 | 1.45E+08 | 1.46E+08 | lincRNA |
| ENSG00000268580 | RP11-514A9.1 | 2 | 1.46E+08 | 1.46E+08 | lincRNA |
| ENSG00000228226 | AC074019.1 | 2 | 2.3E+08 | 2.3E+08 | lincRNA |
| ENSG00000077092 | RARB | 3 | 25215823 | 25639423 | protein_coding |
| ENSG00000164061 | BSN | 3 | 49591922 | 49708978 | protein_coding |
| ENSG00000235120 | BSN-AS1 | 3 | 49677916 | 49679202 | antisense |
| ENSG00000164062 | APEH | 3 | 49711435 | 49721396 | protein_coding |
| ENSG00000173531 | MST1 | 3 | 49721380 | 49726934 | protein_coding |
| ENSG00000259970 | AC099668.5 | 3 | 49721913 | 49722416 | processed_transcript |
| ENSG00000164068 | RNF123 | 3 | 49726932 | 49758962 | protein_coding |
| ENSG00000176020 | AMIGO3 | 3 | 49754267 | 49761349 | protein_coding |
| ENSG00000173540 | GMPPB | 3 | 49754277 | 49761384 | protein_coding |
| ENSG00000176095 | IP6K1 | 3 | 49761727 | 49823975 | protein_coding |
| ENSG00000239576 | COX6CP14 | 3 | 49795916 | 49796089 | pseudogene |
| ENSG00000244730 | RP13-1056D16.2 | 3 | 49812718 | 49813047 | pseudogene |
| ENSG00000187492 | CDHR4 | 3 | 49828165 | 49837268 | protein_coding |
| ENSG00000185614 | FAM212A | 3 | 49840687 | 49842463 | protein_coding |
| ENSG00000182179 | UBA7 | 3 | 49842640 | 49851379 | protein_coding |
| ENSG00000263506 | MIR5193 | 3 | 49843570 | 49843678 | miRNA |
| ENSG00000263651 | AC139451.1 | 3 | 49856387 | 49856486 | miRNA |
| ENSG00000183763 | TRAIP | 3 | 49866034 | 49894007 | protein_coding |
| ENSG00000164076 | CAMKV | 3 | 49895421 | 49907655 | protein_coding |
| ENSG00000264706 | RN7SL217P | 3 | 49900819 | 49901093 | misc_RNA |
| ENSG00000241383 | RP11-152C17.1 | 3 | 1.69E+08 | 1.69E+08 | lincRNA |
| ENSG00000085276 | MECOM | 3 | 1.69E+08 | 1.69E+08 | protein_coding |
| ENSG00000196526 | AFAP1 | 4 | 7760441 | 7941653 | protein_coding |
| ENSG00000138640 | FAM13A | 4 | 89647106 | 90032549 | protein_coding |
| ENSG00000241722 | RP11-255I10.1 | 4 | 1.12E+08 | 1.12E+08 | pseudogene |
| ENSG00000196782 | MAML3 | 4 | 1.41E+08 | 1.41E+08 | protein_coding |
| ENSG00000261129 | RP11-361D14.2 | 4 | 1.45E+08 | 1.45E+08 | lincRNA |
| ENSG00000250504 | KRT18P51 | 4 | 1.45E+08 | 1.45E+08 | pseudogene |
| ENSG00000070193 | FGF10 | 5 | 44303646 | 44389808 | protein_coding |
| ENSG00000248464 | FGF10-AS1 | 5 | 44388834 | 44414091 | antisense |
| ENSG00000164400 | CSF2 | 5 | 1.31E+08 | 1.31E+08 | protein_coding |
| ENSG00000253067 | snoZ6 | 5 | 1.31E+08 | 1.31E+08 | snoRNA |
| ENSG00000072682 | P4HA2 | 5 | 1.32E+08 | 1.32E+08 | protein_coding |
| ENSG00000131435 | PDLIM4 | 5 | 1.32E+08 | 1.32E+08 | protein_coding |
| ENSG00000197208 | SLC22A4 | 5 | 1.32E+08 | 1.32E+08 | protein_coding |
| ENSG00000233006 | AC034220.3 | 5 | 1.32E+08 | 1.32E+08 | processed_transcript |
| ENSG00000263597 | MIR3936 | 5 | 1.32E+08 | 1.32E+08 | miRNA |
| ENSG00000197375 | SLC22A5 | 5 | 1.32E+08 | 1.32E+08 | protein_coding |
| ENSG00000197536 | C5orf56 | 5 | 1.32E+08 | 1.32E+08 | protein_coding |
| ENSG00000238160 | AC116366.5 | 5 | 1.32E+08 | 1.32E+08 | antisense |
| ENSG00000202533 | Y_RNA | 5 | 1.32E+08 | 1.32E+08 | misc_RNA |
| ENSG00000234290 | AC116366.6 | 5 | 1.32E+08 | 1.32E+08 | antisense |
| ENSG00000125347 | IRF1 | 5 | 1.32E+08 | 1.32E+08 | protein_coding |
| ENSG00000164270 | HTR4 | 5 | 1.48E+08 | 1.48E+08 | protein_coding |
| ENSG00000009844 | VTA1 | 6 | 1.42E+08 | 1.43E+08 | protein_coding |
| ENSG00000106261 | ZKSCAN1 | 7 | 99613204 | 99639312 | protein_coding |
| ENSG00000166529 | ZSCAN21 | 7 | 99647390 | 99662661 | protein_coding |
| ENSG00000166526 | ZNF3 | 7 | 99661656 | 99680171 | protein_coding |
| ENSG00000168090 | COPS6 | 7 | 99686577 | 99689823 | protein_coding |
| ENSG00000166508 | MCM7 | 7 | 99690351 | 99699563 | protein_coding |
| ENSG00000207547 | MIR25 | 7 | 99691183 | 99691266 | miRNA |
| ENSG00000207757 | MIR93 | 7 | 99691391 | 99691470 | miRNA |
| ENSG00000208036 | MIR106B | 7 | 99691616 | 99691697 | miRNA |
| ENSG00000221838 | AP4M1 | 7 | 99699172 | 99707968 | protein_coding |
| ENSG00000106290 | TAF6 | 7 | 99704693 | 99717464 | protein_coding |
| ENSG00000242798 | RP11-506M12.1 | 7 | 99712837 | 99724762 | antisense |
| ENSG00000166997 | CNPY4 | 7 | 99717236 | 99723134 | protein_coding |
| ENSG00000214309 | MBLAC1 | 7 | 99724317 | 99726118 | protein_coding |
| ENSG00000235077 | AC073842.19 | 7 | 99728587 | 99738062 | antisense |
| ENSG00000214142 | RPL7P60 | 7 | 99737252 | 99738305 | pseudogene |
| ENSG00000188186 | LAMTOR4 | 7 | 99746530 | 99753567 | protein_coding |
| ENSG00000146826 | C7orf43 | 7 | 99752043 | 99756338 | protein_coding |
| ENSG00000266154 | MIR4658 | 7 | 99754228 | 99754292 | miRNA |
| ENSG00000197093 | GAL3ST4 | 7 | 99756867 | 99766373 | protein_coding |
| ENSG00000213420 | GPC2 | 7 | 99767229 | 99774995 | protein_coding |
| ENSG00000066923 | STAG3 | 7 | 99775186 | 99819111 | protein_coding |
| ENSG00000239521 | GATS | 7 | 99798276 | 99869837 | pseudogene |
| ENSG00000160844 | GATS | 7 | 99798283 | 99869855 | protein_coding |
| ENSG00000213413 | PVRIG | 7 | 99815864 | 99819113 | protein_coding |
| ENSG00000222482 | AC005071.1 | 7 | 99817650 | 99817743 | miRNA |
| ENSG00000078319 | PMS2P1 | 7 | 99918615 | 99939531 | pseudogene |
| ENSG00000201913 | Y_RNA | 7 | 99928400 | 99928500 | misc_RNA |
| ENSG00000242294 | STAG3L5P | 7 | 99933702 | 99949523 | pseudogene |
| ENSG00000272752 | STAG3L5P-PVRIG2P-PILRB | 7 | 99933727 | 99965454 | processed_transcript |
| ENSG00000121716 | PILRB | 7 | 99933737 | 99965356 | protein_coding |
| ENSG00000085514 | PILRA | 7 | 99965153 | 99997719 | protein_coding |
| ENSG00000078487 | ZCWPW1 | 7 | 99998449 | 1E+08 | protein_coding |
| ENSG00000241357 | RP11-758P17.2 | 7 | 1E+08 | 1E+08 | antisense |
| ENSG00000160813 | PPP1R35 | 7 | 1E+08 | 1E+08 | protein_coding |
| ENSG00000240211 | RP11-758P17.3 | 7 | 1E+08 | 1E+08 | antisense |
| ENSG00000166925 | TSC22D4 | 7 | 1E+08 | 1E+08 | protein_coding |
| ENSG00000266372 | AC092849.1 | 7 | 1E+08 | 1E+08 | miRNA |
| ENSG00000166924 | NYAP1 | 7 | 1E+08 | 1E+08 | protein_coding |
| ENSG00000105989 | WNT2 | 7 | 1.17E+08 | 1.17E+08 | protein_coding |
| ENSG00000238202 | AC002465.2 | 7 | 1.17E+08 | 1.17E+08 | antisense |
| ENSG00000154438 | ASZ1 | 7 | 1.17E+08 | 1.17E+08 | protein_coding |
| ENSG00000001626 | CFTR | 7 | 1.17E+08 | 1.17E+08 | protein_coding |
| ENSG00000237974 | AC000111.4 | 7 | 1.17E+08 | 1.17E+08 | pseudogene |
| ENSG00000232661 | AC000111.3 | 7 | 1.17E+08 | 1.17E+08 | antisense |
| ENSG00000234001 | AC000111.5 | 7 | 1.17E+08 | 1.17E+08 | pseudogene |
| ENSG00000083622 | AC000111.6 | 7 | 1.17E+08 | 1.17E+08 | antisense |
| ENSG00000077063 | CTTNBP2 | 7 | 1.17E+08 | 1.18E+08 | protein_coding |
| ENSG00000183638 | RP1L1 | 8 | 10463859 | 10569697 | protein_coding |
| ENSG00000171056 | SOX7 | 8 | 10581278 | 10697357 | protein_coding |
| ENSG00000258724 | SOX7 | 8 | 10582909 | 10697357 | protein_coding |
| ENSG00000248896 | CTD-2135J3.3 | 8 | 10586824 | 10628902 | antisense |
| ENSG00000254093 | PINX1 | 8 | 10622473 | 10697394 | protein_coding |
| ENSG00000252565 | SNORD112 | 8 | 10641565 | 10641629 | snoRNA |
| ENSG00000253695 | RP11-177H2.2 | 8 | 10698086 | 10704011 | antisense |
| ENSG00000171044 | XKR6 | 8 | 10753555 | 11058875 | protein_coding |
| ENSG00000269918 | AF131215.9 | 8 | 10962201 | 10964214 | sense_intronic |
| ENSG00000255310 | AF131215.2 | 8 | 10965298 | 10967236 | sense_intronic |
| ENSG00000254936 | AF131215.3 | 8 | 10980891 | 10983574 | sense_intronic |
| ENSG00000215346 | AF131215.5 | 8 | 10983980 | 10987745 | protein_coding |
| ENSG00000231965 | AF131215.1 | 8 | 11032560 | 11033079 | pseudogene |
| ENSG00000170983 | LINC00208 | 8 | 11433822 | 11438851 | lincRNA |
| ENSG00000120899 | PTK2B | 8 | 27168999 | 27316903 | protein_coding |
| ENSG00000120903 | CHRNA2 | 8 | 27317279 | 27337400 | protein_coding |
| ENSG00000120915 | EPHX2 | 8 | 27348296 | 27403081 | protein_coding |
| ENSG00000234770 | GULOP | 8 | 27417791 | 27446590 | pseudogene |
| ENSG00000120885 | CLU | 8 | 27454434 | 27472548 | protein_coding |
| ENSG00000119487 | MAPKAP1 | 9 | 1.28E+08 | 1.28E+08 | protein_coding |
| ENSG00000229582 | RP11-423C15.3 | 9 | 1.29E+08 | 1.29E+08 | lincRNA |
| ENSG00000167081 | PBX3 | 9 | 1.29E+08 | 1.29E+08 | protein_coding |
| ENSG00000196990 | FAM163B | 9 | 1.36E+08 | 1.36E+08 | protein_coding |
| ENSG00000261018 | LL09NC01-254D11.1 | 9 | 1.36E+08 | 1.36E+08 | antisense |
| ENSG00000158636 | C11orf30 | 11 | 76155967 | 76264069 | protein_coding |
| ENSG00000254755 | RP11-672A2.7 | 11 | 76302067 | 76302255 | pseudogene |
| ENSG00000255970 | RP11-43N5.1 | 12 | 68102827 | 68123255 | lincRNA |
| ENSG00000111249 | CUX2 | 12 | 1.11E+08 | 1.12E+08 | protein_coding |
| ENSG00000221259 | AC002978.1 | 12 | 1.12E+08 | 1.12E+08 | miRNA |
| ENSG00000221386 | AC002979.1 | 12 | 1.12E+08 | 1.12E+08 | miRNA |
| ENSG00000253080 | RNA5SP373 | 12 | 1.12E+08 | 1.12E+08 | rRNA |
| ENSG00000198324 | FAM109A | 12 | 1.12E+08 | 1.12E+08 | protein_coding |
| ENSG00000257595 | RP3-473L9.4 | 12 | 1.12E+08 | 1.12E+08 | lincRNA |
| ENSG00000257539 | HSPA8P14 | 12 | 1.12E+08 | 1.12E+08 | pseudogene |
| ENSG00000111252 | SH2B3 | 12 | 1.12E+08 | 1.12E+08 | protein_coding |
| ENSG00000204842 | ATXN2 | 12 | 1.12E+08 | 1.12E+08 | protein_coding |
| ENSG00000266269 | AC002395.1 | 12 | 1.12E+08 | 1.12E+08 | miRNA |
| ENSG00000272215 | U7 | 12 | 1.12E+08 | 1.12E+08 | snRNA |
| ENSG00000238168 | RP11-686G8.1 | 12 | 1.12E+08 | 1.12E+08 | pseudogene |
| ENSG00000089234 | BRAP | 12 | 1.12E+08 | 1.12E+08 | protein_coding |
| ENSG00000258359 | PCNPP1 | 12 | 1.12E+08 | 1.12E+08 | pseudogene |
| ENSG00000111271 | ACAD10 | 12 | 1.12E+08 | 1.12E+08 | protein_coding |
| ENSG00000257767 | RP11-162P23.2 | 12 | 1.12E+08 | 1.12E+08 | protein_coding |
| ENSG00000111275 | ALDH2 | 12 | 1.12E+08 | 1.12E+08 | protein_coding |
| ENSG00000257877 | RP3-462E2.3 | 12 | 1.12E+08 | 1.12E+08 | lincRNA |
| ENSG00000234608 | MAPKAPK5-AS1 | 12 | 1.12E+08 | 1.12E+08 | lincRNA |
| ENSG00000248594 | AC003029.1 | 12 | 1.12E+08 | 1.12E+08 | pseudogene |
| ENSG00000089022 | MAPKAPK5 | 12 | 1.12E+08 | 1.12E+08 | protein_coding |
| ENSG00000244060 | RPS2P41 | 12 | 1.12E+08 | 1.12E+08 | pseudogene |
| ENSG00000270018 | RP3-462E2.5 | 12 | 1.12E+08 | 1.12E+08 | lincRNA |
| ENSG00000229186 | ADAM1A | 12 | 1.12E+08 | 1.12E+08 | pseudogene |
| ENSG00000226469 | ADAM1B | 12 | 1.12E+08 | 1.12E+08 | pseudogene |
| ENSG00000198270 | TMEM116 | 12 | 1.12E+08 | 1.12E+08 | protein_coding |
| ENSG00000258373 | SLC25A3P2 | 12 | 1.12E+08 | 1.12E+08 | pseudogene |
| ENSG00000257624 | RP1-128M12.3 | 12 | 1.12E+08 | 1.12E+08 | pseudogene |
| ENSG00000089248 | ERP29 | 12 | 1.12E+08 | 1.12E+08 | protein_coding |
| ENSG00000111300 | NAA25 | 12 | 1.12E+08 | 1.13E+08 | protein_coding |
| ENSG00000266370 | MIR3657 | 12 | 1.12E+08 | 1.12E+08 | miRNA |
| ENSG00000258323 | RP1-267L14.3 | 12 | 1.13E+08 | 1.13E+08 | antisense |
| ENSG00000200688 | Y_RNA | 12 | 1.13E+08 | 1.13E+08 | misc_RNA |
| ENSG00000135148 | TRAFD1 | 12 | 1.13E+08 | 1.13E+08 | protein_coding |
| ENSG00000200135 | Y_RNA | 12 | 1.13E+08 | 1.13E+08 | misc_RNA |
| ENSG00000173064 | HECTD4 | 12 | 1.13E+08 | 1.13E+08 | protein_coding |
| ENSG00000257494 | RP3-521E19.2 | 12 | 1.13E+08 | 1.13E+08 | antisense |
| ENSG00000201428 | RN7SKP71 | 12 | 1.13E+08 | 1.13E+08 | misc_RNA |
| ENSG00000213152 | RPL7AP60 | 12 | 1.13E+08 | 1.13E+08 | pseudogene |
| ENSG00000257658 | RP3-521E19.3 | 12 | 1.13E+08 | 1.13E+08 | pseudogene |
| ENSG00000089009 | RPL6 | 12 | 1.13E+08 | 1.13E+08 | protein_coding |
| ENSG00000179295 | PTPN11 | 12 | 1.13E+08 | 1.13E+08 | protein_coding |
| ENSG00000119698 | PPP4R4 | 14 | 94612465 | 94746072 | protein_coding |
| ENSG00000140093 | SERPINA10 | 14 | 94749650 | 94759608 | protein_coding |
| ENSG00000258597 | SERPINA2P | 14 | 94830053 | 94833039 | pseudogene |
| ENSG00000197249 | SERPINA1 | 14 | 94843084 | 94857030 | protein_coding |
| ENSG00000138593 | SECISBP2L | 15 | 49280673 | 49338760 | protein_coding |
| ENSG00000207105 | Y_RNA | 15 | 49288109 | 49288219 | misc_RNA |
| ENSG00000166200 | COPS2 | 15 | 49398268 | 49447858 | protein_coding |
| ENSG00000200120 | Y_RNA | 15 | 49429959 | 49430060 | misc_RNA |
| ENSG00000156958 | GALK2 | 15 | 49447853 | 49660066 | protein_coding |
| ENSG00000259467 | NDUFAF4P1 | 15 | 49448526 | 49449044 | pseudogene |
| ENSG00000264210 | MIR4716 | 15 | 49461267 | 49461350 | miRNA |
| ENSG00000259545 | RP11-325E5.4 | 15 | 49469807 | 49470938 | pseudogene |
| ENSG00000243338 | RN7SL307P | 15 | 49491099 | 49491391 | misc_RNA |
| ENSG00000166262 | FAM227B | 15 | 49619159 | 49913128 | protein_coding |
| ENSG00000259531 | RP11-295H24.3 | 15 | 49657321 | 49658882 | pseudogene |
| ENSG00000140285 | FGF7 | 15 | 49715293 | 49780972 | protein_coding |
| ENSG00000104047 | DTWD1 | 15 | 49913177 | 49937333 | protein_coding |
| ENSG00000261597 | RP11-353B9.1 | 15 | 49944336 | 49948429 | sense_overlapping |
| ENSG00000166949 | SMAD3 | 15 | 67356101 | 67487533 | protein_coding |
| ENSG00000103591 | AAGAB | 15 | 67493371 | 67547533 | protein_coding |
| ENSG00000242634 | RPS24P16 | 15 | 67524784 | 67525175 | pseudogene |
| ENSG00000103599 | IQCH | 15 | 67547138 | 67794598 | protein_coding |
| ENSG00000187720 | THSD4 | 15 | 71389291 | 72075722 | protein_coding |
| ENSG00000212664 | RP11-592N21.1 | 15 | 71633466 | 71634086 | pseudogene |
| ENSG00000260586 | RP11-592N21.2 | 15 | 71634663 | 71640810 | sense_intronic |
| ENSG00000136381 | IREB2 | 15 | 78729773 | 78793798 | protein_coding |
| ENSG00000259474 | RP11-650L12.1 | 15 | 78772399 | 78774162 | antisense |
| ENSG00000188266 | HYKK | 15 | 78799906 | 78829714 | protein_coding |
| ENSG00000268838 | AC027228.1 | 15 | 78830023 | 78831288 | protein_coding |
| ENSG00000041357 | PSMA4 | 15 | 78832747 | 78841604 | protein_coding |
| ENSG00000169684 | CHRNA5 | 15 | 78857862 | 78887611 | protein_coding |
| ENSG00000261762 | RP11-650L12.2 | 15 | 78881465 | 78883618 | antisense |
| ENSG00000080644 | CHRNA3 | 15 | 78885394 | 78913637 | protein_coding |
| ENSG00000117971 | CHRNB4 | 15 | 78916461 | 79020096 | protein_coding |
| ENSG00000259555 | RP11-335K5.2 | 15 | 78918237 | 78920535 | antisense |
| ENSG00000261303 | RP11-160C18.2 | 15 | 78952986 | 79027837 | pseudogene |
| ENSG00000213307 | RPL18P11 | 15 | 78953389 | 78953918 | pseudogene |
| ENSG00000238166 | RP11-160C18.4 | 15 | 79044449 | 79045715 | pseudogene |
| ENSG00000136378 | ADAMTS7 | 15 | 79051545 | 79103773 | protein_coding |
| ENSG00000185787 | MORF4L1 | 15 | 79102829 | 79190475 | protein_coding |
| ENSG00000243711 | RPL21P116 | 15 | 79155589 | 79156071 | pseudogene |
| ENSG00000048471 | SNX29 | 16 | 12070594 | 12668146 | protein_coding |
| ENSG00000260601 | RP11-552C15.1 | 16 | 12639339 | 12640541 | sense_intronic |
| ENSG00000259876 | CTD-3037G24.4 | 16 | 12650210 | 12651551 | antisense |
| ENSG00000259899 | CTD-3037G24.3 | 16 | 12654613 | 12704901 | antisense |
| ENSG00000171532 | NEUROD2 | 17 | 37759789 | 37766030 | protein_coding |
| ENSG00000214546 | AC087491.2 | 17 | 37775866 | 37778766 | lincRNA |
| ENSG00000131771 | PPP1R1B | 17 | 37782993 | 37792879 | protein_coding |
| ENSG00000131748 | STARD3 | 17 | 37793318 | 37819737 | protein_coding |
| ENSG00000173991 | TCAP | 17 | 37820440 | 37822808 | protein_coding |
| ENSG00000141744 | PNMT | 17 | 37824234 | 37826728 | protein_coding |
| ENSG00000161395 | PGAP3 | 17 | 37827375 | 37853050 | protein_coding |
| ENSG00000141736 | ERBB2 | 17 | 37844167 | 37886679 | protein_coding |
| ENSG00000265178 | MIR4728 | 17 | 37882748 | 37882814 | miRNA |
| ENSG00000141741 | MIEN1 | 17 | 37884749 | 37887040 | protein_coding |
| ENSG00000141738 | GRB7 | 17 | 37894180 | 37903544 | protein_coding |
| ENSG00000264198 | RP11-94L15.2 | 17 | 37913974 | 37920089 | lincRNA |
| ENSG00000161405 | IKZF3 | 17 | 37921198 | 38020441 | protein_coding |
| ENSG00000264663 | KRT8P34 | 17 | 37991290 | 37992333 | pseudogene |
| ENSG00000226117 | AC079199.2 | 17 | 37995622 | 37995777 | pseudogene |
| ENSG00000186075 | ZPBP2 | 17 | 38024417 | 38034149 | protein_coding |
| ENSG00000073605 | GSDMB | 17 | 38060848 | 38076107 | protein_coding |
| ENSG00000172057 | ORMDL3 | 17 | 38077294 | 38083854 | protein_coding |
| ENSG00000264968 | RP11-387H17.4 | 17 | 38083995 | 38095854 | lincRNA |
| ENSG00000204913 | LRRC3C | 17 | 38097727 | 38101000 | protein_coding |
| ENSG00000008838 | MED24 | 17 | 38175350 | 38217468 | protein_coding |
| ENSG00000238793 | SNORD124 | 17 | 38183795 | 38183898 | snoRNA |
| ENSG00000126351 | THRA | 17 | 38214543 | 38250120 | protein_coding |
| ENSG00000268797 | CTC-490E21.12 | 19 | 41307202 | 41404187 | protein_coding |
| ENSG00000237118 | CYP2F2P | 19 | 41324626 | 41332677 | pseudogene |
| ENSG00000269843 | CTC-490E21.10 | 19 | 41337126 | 41343115 | lincRNA |
| ENSG00000255974 | CYP2A6 | 19 | 41349443 | 41356352 | protein_coding |
| ENSG00000198077 | CYP2A7 | 19 | 41381344 | 41388657 | protein_coding |
| ENSG00000130612 | CYP2G1P | 19 | 41396731 | 41406413 | pseudogene |
| ENSG00000198251 | CTC-490E21.13 | 19 | 41414377 | 41416754 | pseudogene |
| ENSG00000256612 | CYP2B7P | 19 | 41430124 | 41456565 | pseudogene |
| ENSG00000130202 | PVRL2 | 19 | 45349432 | 45392485 | protein_coding |
| ENSG00000267282 | CTB-129P6.4 | 19 | 45385284 | 45394133 | antisense |
| ENSG00000130204 | TOMM40 | 19 | 45393826 | 45406946 | protein_coding |
| ENSG00000130203 | APOE | 19 | 45409011 | 45412650 | protein_coding |
| ENSG00000130208 | APOC1 | 19 | 45417504 | 45422606 | protein_coding |
| ENSG00000214855 | APOC1P1 | 19 | 45430061 | 45434643 | pseudogene |
| ENSG00000212314 | RNU6-1307P | 19 | 54767805 | 54767913 | snRNA |
| ENSG00000240042 | AC098789.1 | 19 | 54771858 | 54772896 | pseudogene |
| ENSG00000240296 | AC010492.5 | 19 | 54771859 | 54772899 | pseudogene |
| ENSG00000131042 | LILRB2 | 19 | 54777675 | 54785039 | protein_coding |
| ENSG00000264703 | MIR4752 | 19 | 54785964 | 54786035 | miRNA |
| ENSG00000240197 | AC010518.3 | 19 | 54797902 | 54798219 | pseudogene |
| ENSG00000170866 | LILRA3 | 19 | 54799854 | 54809952 | protein_coding |
| ENSG00000251431 | AC008984.5 | 19 | 54808714 | 54809423 | pseudogene |
| ENSG00000101204 | CHRNA4 | 20 | 61975420 | 62009753 | protein_coding |
| ENSG00000203900 | RP11-261N11.8 | 20 | 61991340 | 62002529 | antisense |

ENSG, ensembl gene; CHR, chromosome

**Table S7** Annotation of genomic loci with a p-value less than 5e-08 in the context of COPD.

| **ENSG** | **SYMBOL** | **CHR** | **START** | **END** | **Type** |
| --- | --- | --- | --- | --- | --- |
| ENSG00000164362 | TERT | 5 | 1253262 | 1295184 | protein_coding |
| ENSG00000129595 | EPB41L4A | 5 | 1.11E+08 | 1.12E+08 | protein_coding |
| ENSG00000248430 | HMGB3P16 | 5 | 1.12E+08 | 1.12E+08 | pseudogene |
| ENSG00000258864 | CTC-554D6.1 | 5 | 1.12E+08 | 1.12E+08 | protein_coding |
| ENSG00000272869 | CTC-487M23.8 | 5 | 1.12E+08 | 1.12E+08 | protein_coding |
| ENSG00000153037 | SRP19 | 5 | 1.12E+08 | 1.12E+08 | protein_coding |
| ENSG00000129625 | REEP5 | 5 | 1.12E+08 | 1.12E+08 | protein_coding |
| ENSG00000212643 | ZRSR1 | 5 | 1.12E+08 | 1.12E+08 | protein_coding |
| ENSG00000270067 | CTC-487M23.5 | 5 | 1.12E+08 | 1.12E+08 | antisense |
| ENSG00000272389 | CTC-487M23.7 | 5 | 1.12E+08 | 1.12E+08 | antisense |
| ENSG00000266499 | AC008536.1 | 5 | 1.12E+08 | 1.12E+08 | miRNA |
| ENSG00000171444 | MCC | 5 | 1.12E+08 | 1.13E+08 | protein_coding |
| ENSG00000232633 | CTD-2201G3.1 | 5 | 1.13E+08 | 1.13E+08 | antisense |
| ENSG00000212122 | TSSK1B | 5 | 1.13E+08 | 1.13E+08 | protein_coding |
| ENSG00000047188 | YTHDC2 | 5 | 1.13E+08 | 1.13E+08 | protein_coding |
| ENSG00000251628 | RP11-371M22.1 | 5 | 1.13E+08 | 1.13E+08 | lincRNA |
| ENSG00000147677 | EIF3H | 8 | 1.18E+08 | 1.18E+08 | protein_coding |
| ENSG00000246228 | CASC8 | 8 | 1.28E+08 | 1.28E+08 | antisense |
| ENSG00000253929 | RP11-382A18.2 | 8 | 1.28E+08 | 1.28E+08 | lincRNA |
| ENSG00000212993 | POU5F1B | 8 | 1.28E+08 | 1.28E+08 | protein_coding |
| ENSG00000212505 | RNA5SP299 | 10 | 8698679 | 8698794 | rRNA |
| ENSG00000270234 | RP11-575N15.1 | 10 | 8765973 | 8766251 | pseudogene |
| ENSG00000077514 | POLD3 | 11 | 74204896 | 74380162 | protein_coding |
| ENSG00000254500 | RANP3 | 11 | 74363681 | 74363956 | pseudogene |
| ENSG00000054938 | CHRDL2 | 11 | 74407474 | 74442430 | protein_coding |
| ENSG00000185162 | AP001324.1 | 11 | 74409389 | 74409869 | pseudogene |
| ENSG00000212277 | SNORD43 | 11 | 74427732 | 74427794 | snoRNA |
| ENSG00000265902 | MIR4696 | 11 | 74431313 | 74431382 | miRNA |
| ENSG00000166435 | XRRA1 | 11 | 74518784 | 74660245 | protein_coding |
| ENSG00000241170 | RP11-147I3.1 | 11 | 74587331 | 74631067 | pseudogene |
| ENSG00000265344 | AP001992.1 | 11 | 74657026 | 74657123 | miRNA |
| ENSG00000118363 | SPCS2 | 11 | 74660292 | 74690076 | protein_coding |
| ENSG00000150750 | C11orf53 | 11 | 1.11E+08 | 1.11E+08 | protein_coding |
| ENSG00000196167 | COLCA1 | 11 | 1.11E+08 | 1.11E+08 | protein_coding |
| ENSG00000214290 | COLCA2 | 11 | 1.11E+08 | 1.11E+08 | protein_coding |
| ENSG00000050405 | LIMA1 | 12 | 50569571 | 50677329 | protein_coding |
| ENSG00000257298 | RP3-405J10.3 | 12 | 50579363 | 50585146 | sense_intronic |
| ENSG00000257531 | RP3-405J10.2 | 12 | 50611445 | 50612126 | pseudogene |
| ENSG00000257256 | RP3-405J10.4 | 12 | 50613387 | 50623767 | antisense |
| ENSG00000221604 | MIR1293 | 12 | 50627925 | 50627995 | miRNA |
| ENSG00000212496 | RNU6-1093P | 12 | 50650021 | 50650127 | snRNA |
| ENSG00000203433 | AC008147.2 | 12 | 50678449 | 50678545 | pseudogene |
| ENSG00000203432 | AC008147.1 | 12 | 50683977 | 50684073 | pseudogene |
| ENSG00000203431 | AC140061.11 | 12 | 50684744 | 50684840 | pseudogene |
| ENSG00000203430 | AC140061.10 | 12 | 50688917 | 50689013 | pseudogene |
| ENSG00000269529 | AC140061.12 | 12 | 50690489 | 50691718 | protein_coding |
| ENSG00000203428 | AC140061.9 | 12 | 50695577 | 50695673 | pseudogene |
| ENSG00000203427 | AC140061.8 | 12 | 50697570 | 50697669 | pseudogene |
| ENSG00000203426 | AC140061.7 | 12 | 50698425 | 50698530 | pseudogene |
| ENSG00000196082 | AC140061.1 | 12 | 50706112 | 50706217 | pseudogene |
| ENSG00000203425 | AC140061.6 | 12 | 50706430 | 50706526 | pseudogene |
| ENSG00000203424 | AC140061.5 | 12 | 50707798 | 50707894 | pseudogene |
| ENSG00000203423 | AC140061.4 | 12 | 50710006 | 50710100 | pseudogene |
| ENSG00000203422 | AC140061.3 | 12 | 50710653 | 50710749 | pseudogene |
| ENSG00000203421 | AC140061.2 | 12 | 50711462 | 50711558 | pseudogene |
| ENSG00000185958 | FAM186A | 12 | 50720013 | 50790405 | protein_coding |
| ENSG00000244266 | RP11-112N23.1 | 12 | 50759435 | 50759869 | pseudogene |
| ENSG00000161813 | LARP4 | 12 | 50786166 | 50873787 | protein_coding |
| ENSG00000066084 | DIP2B | 12 | 50898768 | 51142450 | protein_coding |
| ENSG00000207136 | RNU6-769P | 12 | 51027056 | 51027159 | snRNA |
| ENSG00000200183 | RNU6-238P | 12 | 51050756 | 51050861 | snRNA |
| ENSG00000200428 | Y_RNA | 12 | 51137351 | 51137467 | misc_RNA |
| ENSG00000271018 | RP11-60E8.2 | 12 | 51150675 | 51151216 | pseudogene |
| ENSG00000123268 | ATF1 | 12 | 51157493 | 51214905 | protein_coding |
| ENSG00000271490 | RP11-60E8.4 | 12 | 51200118 | 51200220 | pseudogene |
| ENSG00000166922 | SCG5 | 15 | 32933877 | 32989299 | protein_coding |
| ENSG00000259721 | RP11-758N13.1 | 15 | 33009471 | 33011208 | lincRNA |
| ENSG00000166923 | GREM1 | 15 | 33010175 | 33026870 | protein_coding |
| ENSG00000175711 | B3GNTL1 | 17 | 80900031 | 81009686 | protein_coding |
| ENSG00000176845 | METRNL | 17 | 81037567 | 81052864 | protein_coding |
| ENSG00000261888 | AC144831.1 | 17 | 81061205 | 81063860 | lincRNA |
| ENSG00000101665 | SMAD7 | 18 | 46446223 | 46477081 | protein_coding |
| ENSG00000131941 | RHPN2 | 19 | 33469499 | 33555794 | protein_coding |
| ENSG00000130702 | LAMA5 | 20 | 60883011 | 60942368 | protein_coding |
| ENSG00000265329 | MIR4758 | 20 | 60907543 | 60907613 | miRNA |
| ENSG00000228812 | RP11-157P1.5 | 20 | 60928051 | 60931536 | antisense |
| ENSG00000171858 | RPS21 | 20 | 60962172 | 60963576 | protein_coding |
| ENSG00000149679 | CABLES2 | 20 | 60963688 | 60982341 | protein_coding |
| ENSG00000130701 | RBBP8NL | 20 | 60985293 | 61002589 | protein_coding |

ENSG, ensembl gene; CHR, chromosome





**Figure S1** Effects of characteristics on risk of colon cancer in NHANES. P value < 0.05 was considered statistically significant.

Abbreviations: OR, odds ratio; CI, confidence intervals; COPD, chronic obstructive pulmonary disease.


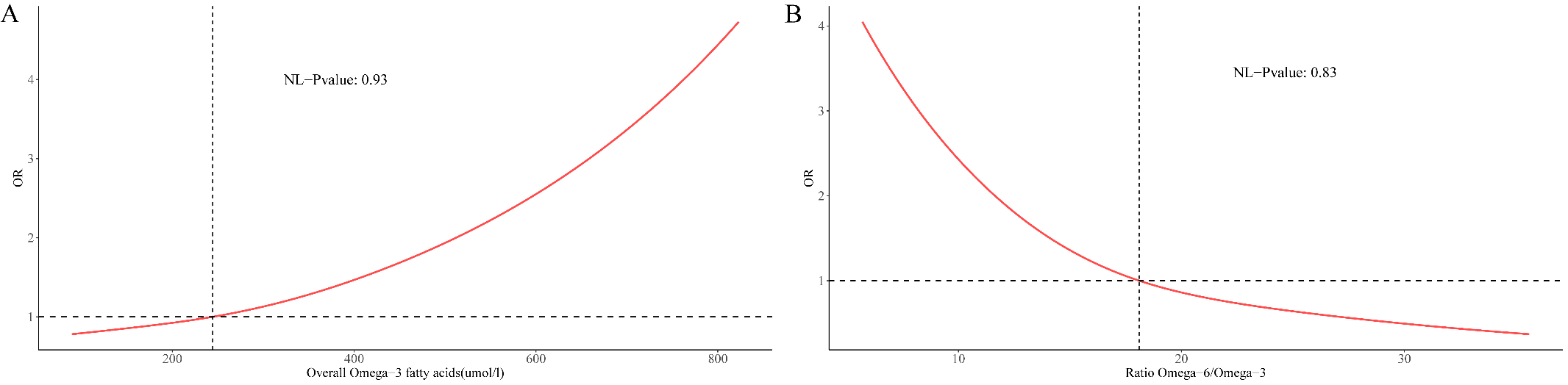


**Figure S2** Dose-response association among overall Omega-3 fatty acids and ratio of overall Omega-6 fatty acids to overall Omega-3 fatty acids with colon cancer. (A)Dose-response association between overall Omega-3 fatty acids and colon cancer; (B)Dose-response association between ratio Omega-6/Omega-3 and colon cancer.


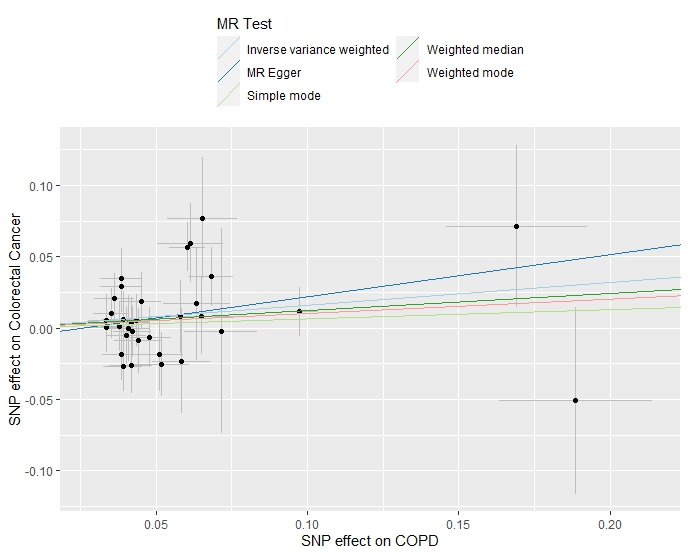


**Figure S3** Causal effects of COPD on CRC.

IVW, inverse variance weighted; MR, Mendelian randomization. The lines denote effect sizes.


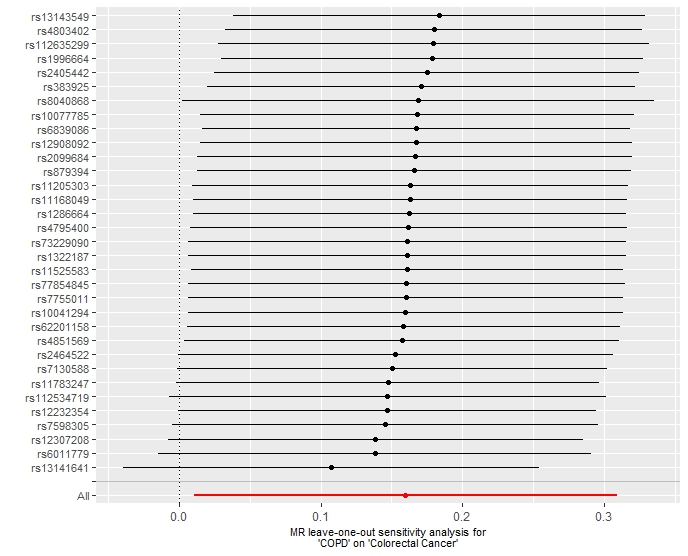


**Figure S4** Leave-one-out analysis for the association of COPD and CRC. This figure shows the results of the leave-one-out analysis for all significant causal relationships identified in the primary analysis. The black dots represent the estimated causal association between a specific exposure and the target mental disorder when each SNP is removed in turn. The red dots represent the overall causal estimate using the random-effects inverse variance weighted method. The horizontal lines indicate the 95% confidence intervals.


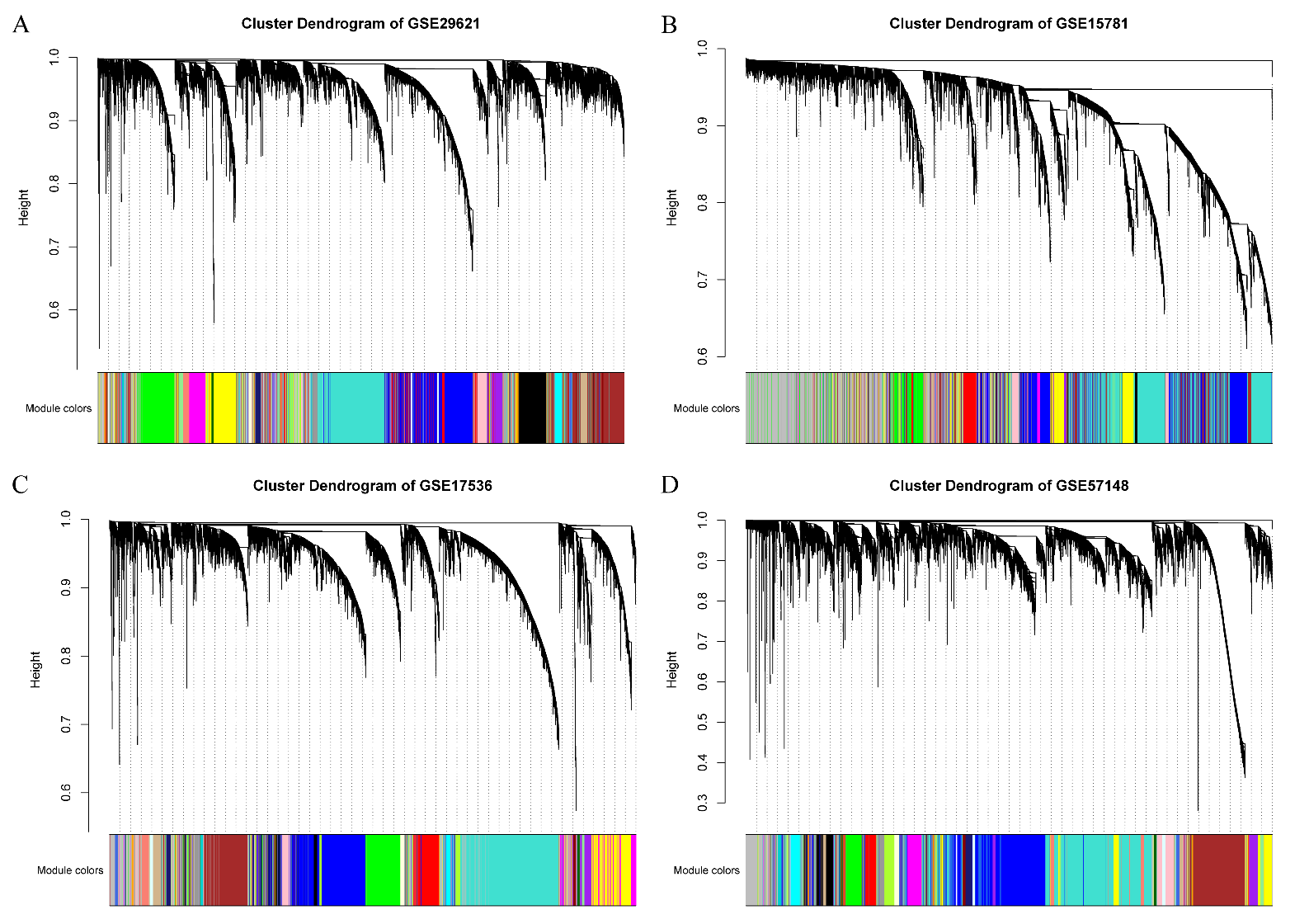


**Figure S5** Cluster Dendrogram of CRC and COPD RNA-seq datasets GSE29621, GSE15781, GSE17536, GSE57148. Each color represents a module where genes with similar expression patterns are clustered together. Longer branches represent lower similarity, while shorter branches indicate higher similarity.
